# Supplementary material for: Multi-ancestry genome-wide meta-analysis identifies novel basal cell carcinoma loci and shared genetic effects with squamous cell carcinoma
Source: Commun Biol. 2024 Jan 5;7:33. doi: 10.1038/s42003-023-05753-7 (PMC10770328; doi:10.1038/s42003-023-05753-7)
Supplement: Supplementary file 2 — Supplementary Information [file 42003_2023_5753_MOESM2_ESM.pdf]

Choquet et al. "Multi-ancestry genome-wide meta-analysis identifies novel basal cell carcinoma loci and shared genetic effects with squamous cell carcinoma"

Supplementary Figures

Supplementary Figure 1. QQ plot and genomic inflation factor (λ) observed for the European ancestry GWA meta-analysis of BCC.

Supplementary Figure 2. Manhattan plot of the European ancestry GWA meta-analysis of BCC.

Supplementary Figure 3. Locus Zoom plots of novel regions identified in the European ancestry GWA meta-analysis of BCC**.**

Supplementary Figure 4. Forest plots of new BCC loci identified in the European ancestry GWA meta-analysis.

Supplementary Figure 5. QQ plot and genomic inflation factor (λ) observed for the Hispanic/Latino ancestry GWA meta-analysis of BCC.

Supplementary Figure 6. Manhattan plot of the Hispanic/Latino ancestry GWA meta-analysis of BCC.

Supplementary Figure 7. Locus Zoom plots of regions identified in the Hispanic/Latino GWA meta-analysis of BCC or showing association with BCC at Bonferroni level of significance.

Supplementary Figure 8. Receiver operating characteristic curves for BCC predictions in the GERA Hispanic/Latino sample, using polygenic risk scores derived from the European ancestry GWA meta-analysis of BCC.

Supplementary Figure 9. QQ plot and genomic inflation factor (λ) observed for the multi-ancestry GWA meta-analysis of BCC.

Supplementary Figure 10. Locus Zoom plots of additional novel regions identified in the multi-ancestry GWA meta-analysis of BCC.

Supplementary Figure 11. Correlation of effect sizes across populations (European ancestry vs. Hispanic/Latino) for the lead 116 BCC-associated lead SNPs identified in the European ancestry GWA meta-analysis of BCC.

Supplementary Figure 12. QQ plot and genomic inflation factor (λ) observed for the European ancestry GWA meta-analysis of SCC.

Supplementary Figure 13. Manhattan plot of the European ancestry GWA meta-analysis of SCC.

Supplementary Figure 14. Genome-wide genetic correlations between BCC and SCC across cohorts.

Supplementary Notes

Supplementary Note 1: Details for UKB on phenotyping

Supplementary Note 2: Details for each cohort on genotyping, imputation, and quality control

Supplementary Figure 1. QQ plot and genomic inflation factor (λ) observed for the European ancestry GWA meta-analysis of BCC

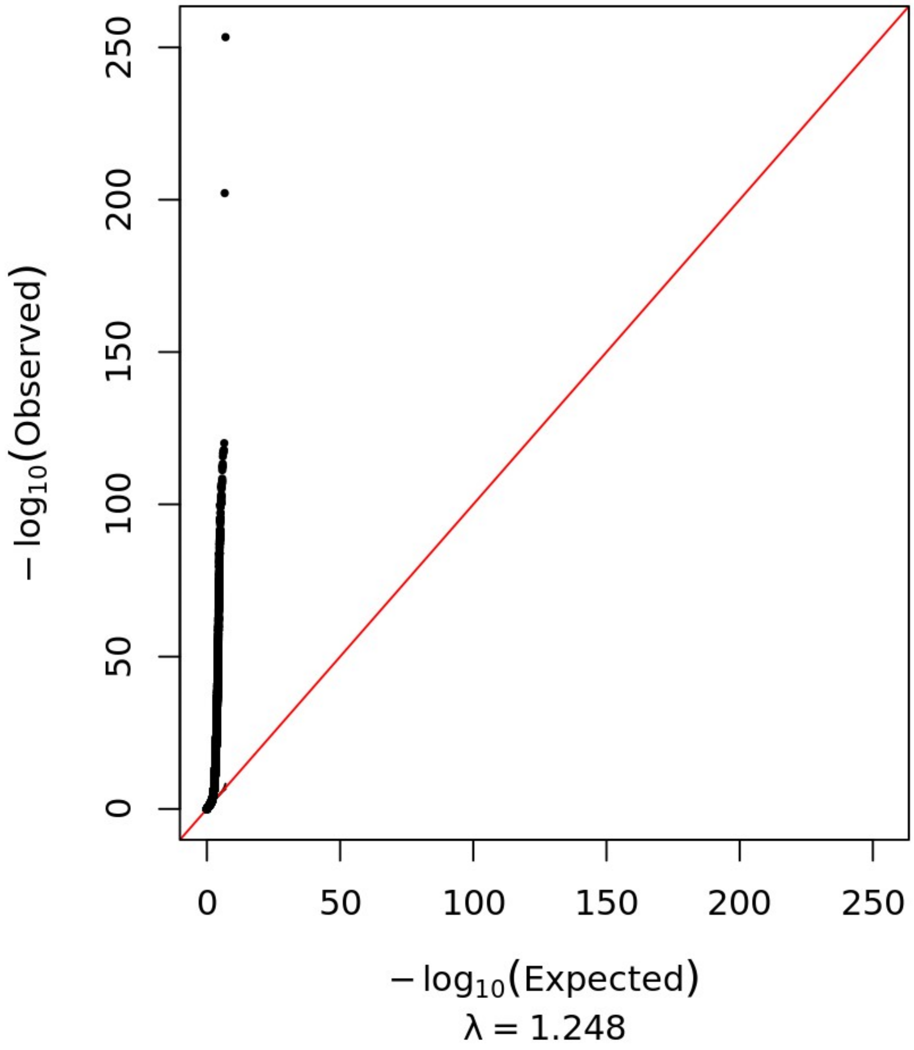

Supplementary Figure 2. Manhattan plot of the European ancestry GWA meta-analysis of BCC. The y-axis represents the -log$_{10}$(P-value); all P-values derived from logistic regression model are two-sided. The red dotted line represents the threshold of P=5x10^{-8} which is the commonly accepted threshold of adjustments for multiple comparisons in GWAS. Locus names in blue are for the novel loci and the ones in dark are for the previously reported ones.

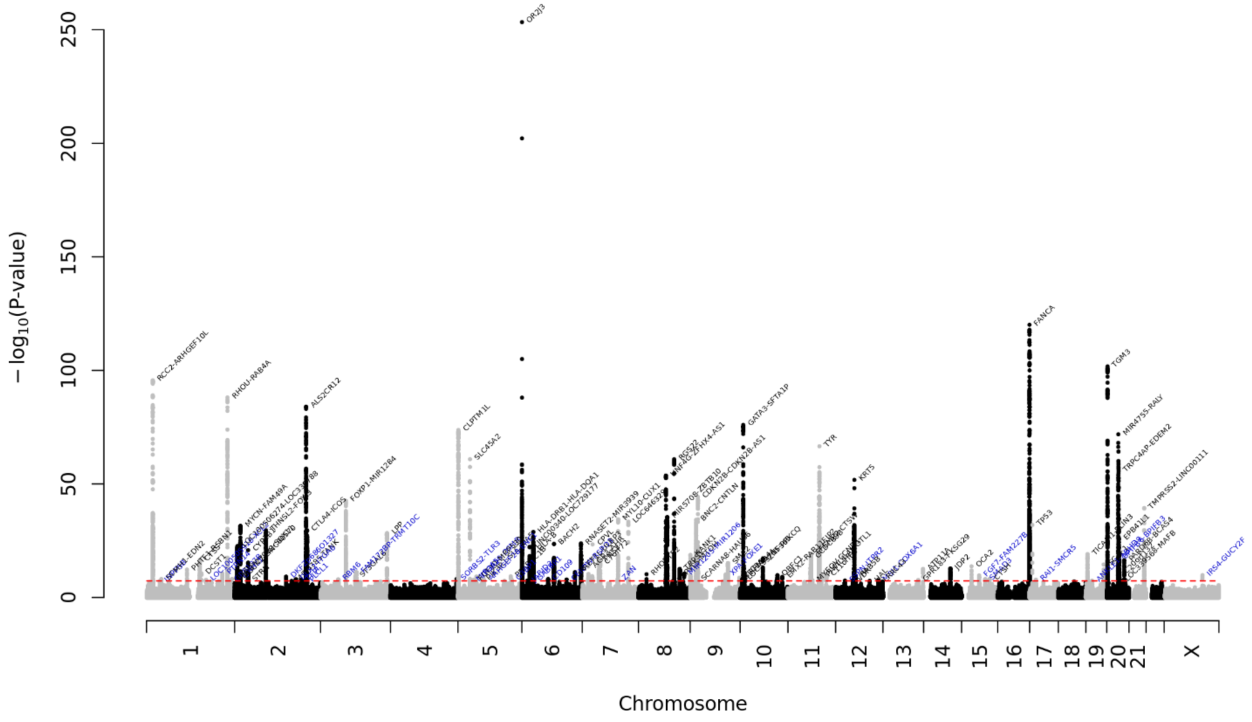

Supplementary Figure 3. Locus Zoom plots of novel regions identified in the European ancestry GWA meta-analysis of BCC**.**

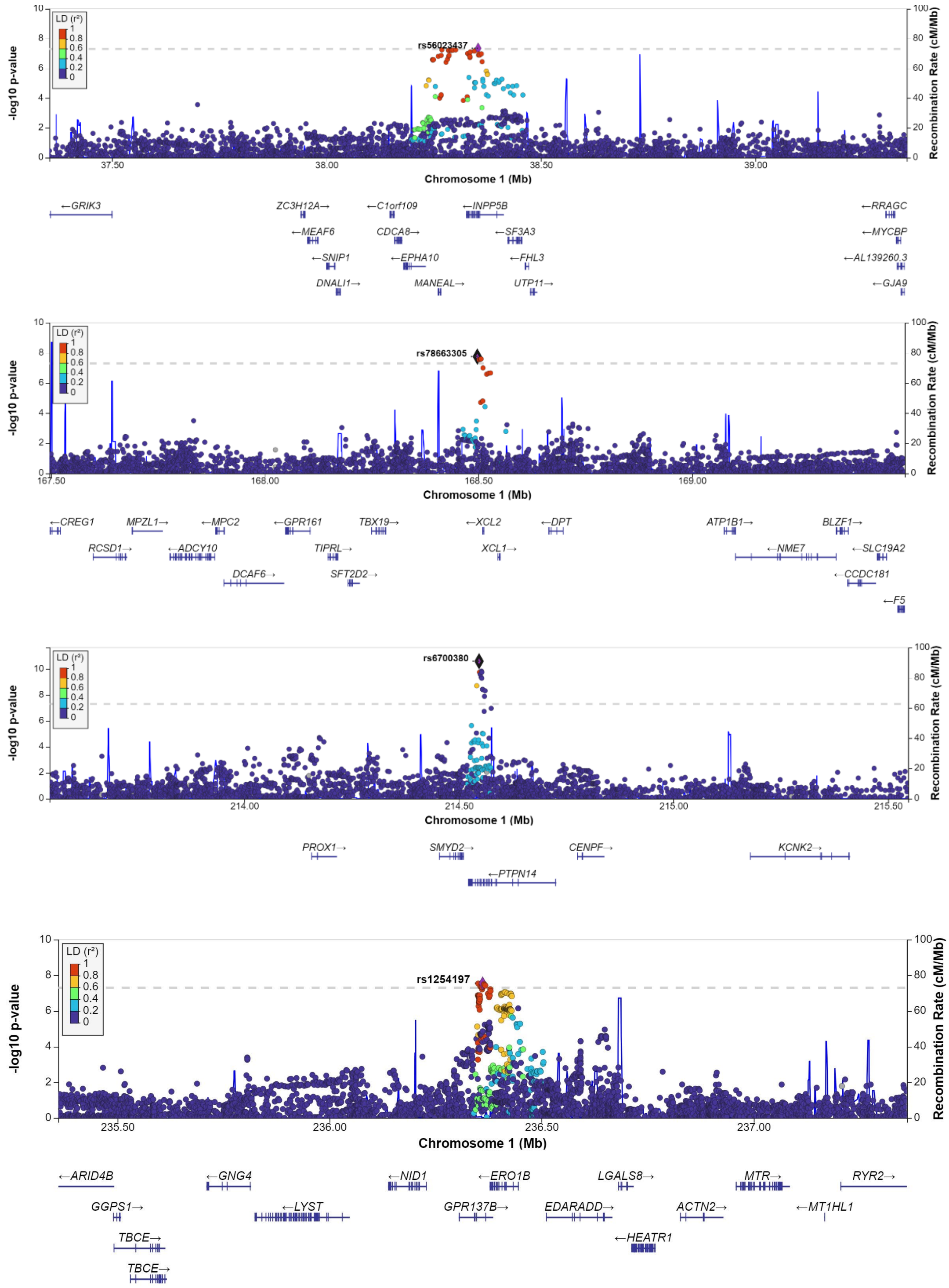

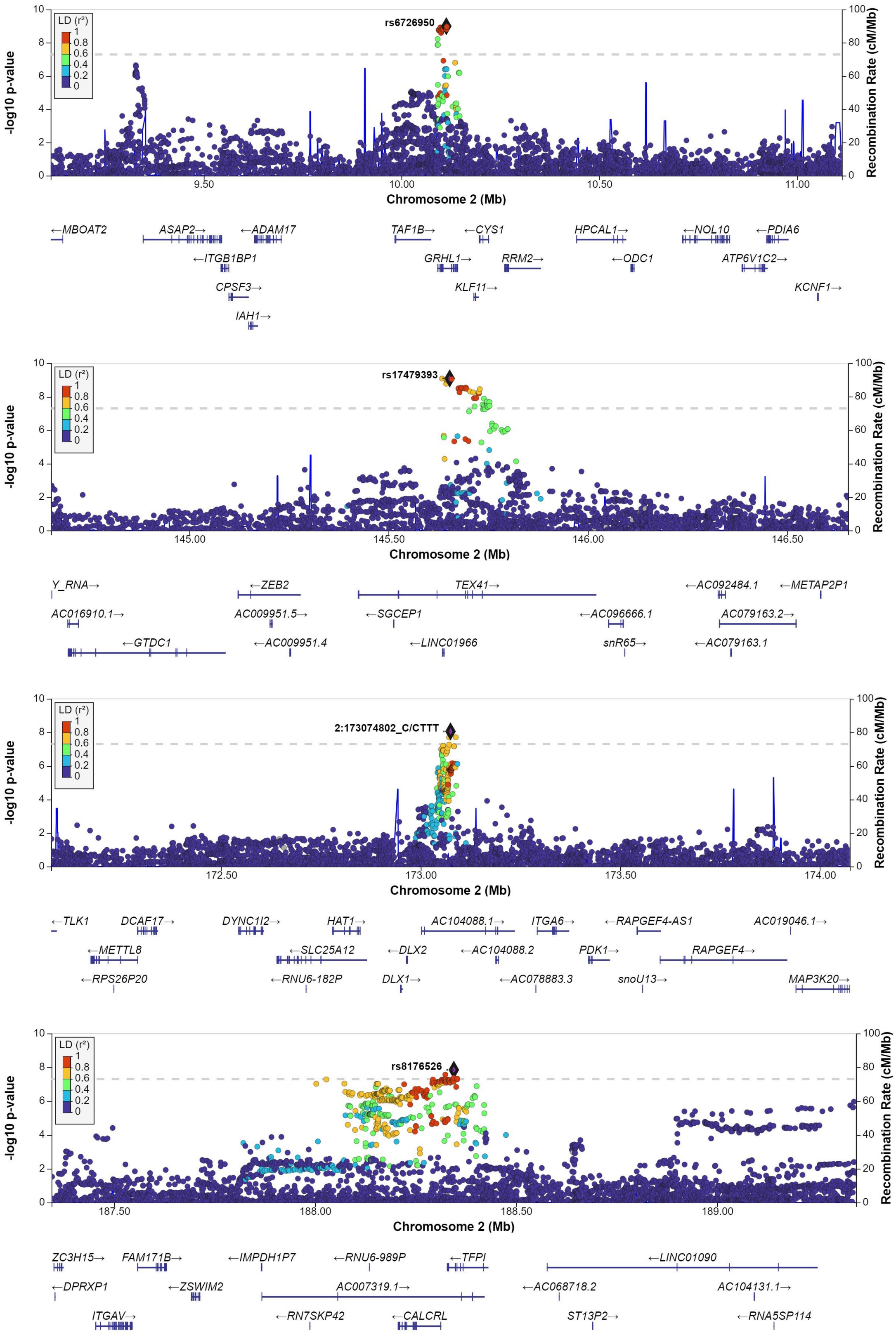

rs6726950
LD (r²)
-log10 p-value
Recombination Rate (cM/Mb)
Chromosome 2 (Mb)
9.50
10.00
10.50
11.00
←MBOAT2
ASAP2→
←ADAM17
←ITGB1BP1
CPSF3→
IAH1→
TAF1B→
←CYS1
GRHL1→
RRM2→
KLF11→
HPCAL1→
←ODC1
←NOL10
←PDIA6
ATP6V1C2→
KCNF1→
rs17479393
145.00
145.50
146.00
146.50
Y_RNA→
AC016910.1→
←GTDC1
←ZEB2
AC009951.5→
←AC009951.4
TEX41→
←SGCEP1
←LINC01966
←AC096666.1
snR65→
←AC092484.1
←METAP2P1
AC079163.2→
←AC079163.1
2:173074802_C/CTTT
172.50
173.00
173.50
174.00
←TLK1
DCAF17→
←METTL8
←RPS26P20
DYNC1I2→
←SLC25A12
←RNU6-182P
HAT1→
←DLX2
DLX1→
AC104088.1→
←AC104088.2
←AC078883.3
ITGA6→
PDK1→
←RAPGEF4-AS1
RAPGEF4→
snoU13→
AC019046.1→
MAP3K20→
rs8176526
187.50
188.00
188.50
189.00
ZC3H15→
←DPRXP1
ITGAV→
FAM171B→
←ZSWIM2
←IMPDH1P7
AC007319.1→
←RN7SKP42
←RNU6-989P
←TFPI
←CALCRL
←AC068718.2
ST13P2→
←LINC01090
AC104131.1→
←RNA5SP114

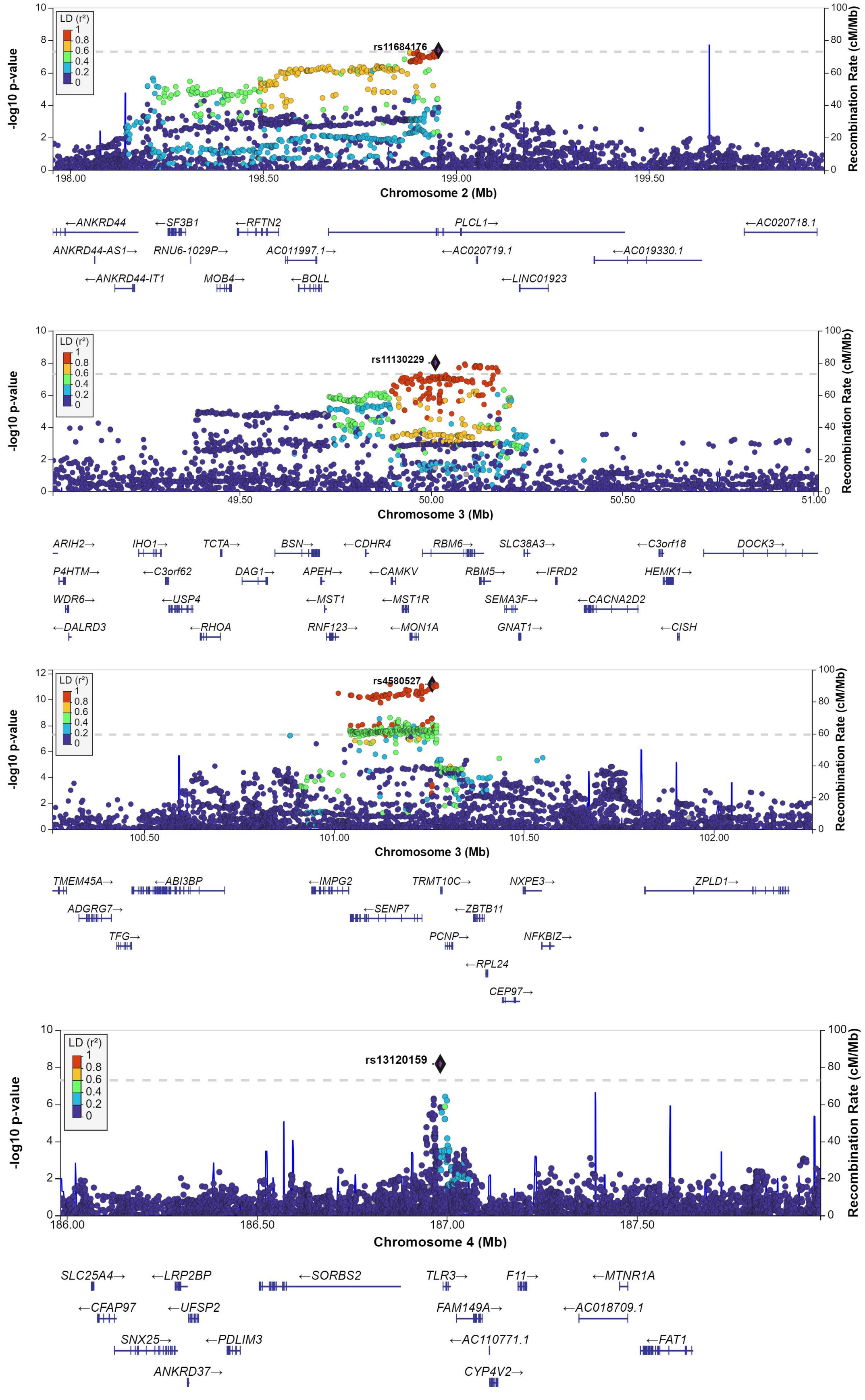

LD (r²)
1
0.8
0.6
0.4
0.2
0
rs11684176
-log10 p-value
Recombination Rate (cM/Mb)
198.00
198.50
199.00
199.50
Chromosome 2 (Mb)
←ANKRD44
←SF3B1
←RFTN2
PLCL1→
←AC020718.1
ANKRD44-AS1→
RNU6-1029P→
AC011997.1→
←AC020719.1
←AC019330.1
←ANKRD44-IT1
MOB4→
←BOLL
←LINC01923
rs11130229
49.50
50.00
50.50
51.00
Chromosome 3 (Mb)
ARIH2→
IHO1→
TCTA→
BSN→
←CDHR4
RBM6→
SLC38A3→
←C3orf18
DOCK3→
P4HTM→
←C3orf62
DAG1→
APEH→
←CAMKV
RBM5→
←IFRD2
HEMK1→
WDR6→
←USP4
←MST1
←MST1R
SEMA3F→
←CACNA2D2
←DALRD3
←RHOA
RNF123→
←MON1A
GNAT1→
←CISH
rs4580527
100.50
101.00
101.50
102.00
Chromosome 3 (Mb)
TMEM45A→
←ABI3BP
←IMPG2
TRMT10C→
NXPE3→
ZPLD1→
ADGRG7→
←SENP7
←ZBTB11
TFG→
PCNP→
NFKBIZ→
←RPL24
CEP97→
rs13120159
186.00
186.50
187.00
187.50
Chromosome 4 (Mb)
SLC25A4→
←LRP2BP
←SORBS2
TLR3→
F11→
←MTNR1A
←CFAP97
←UFSP2
FAM149A→
←AC018709.1
SNX25→
←PDLIM3
←AC110771.1
←FAT1
ANKRD37→
CYP4V2→

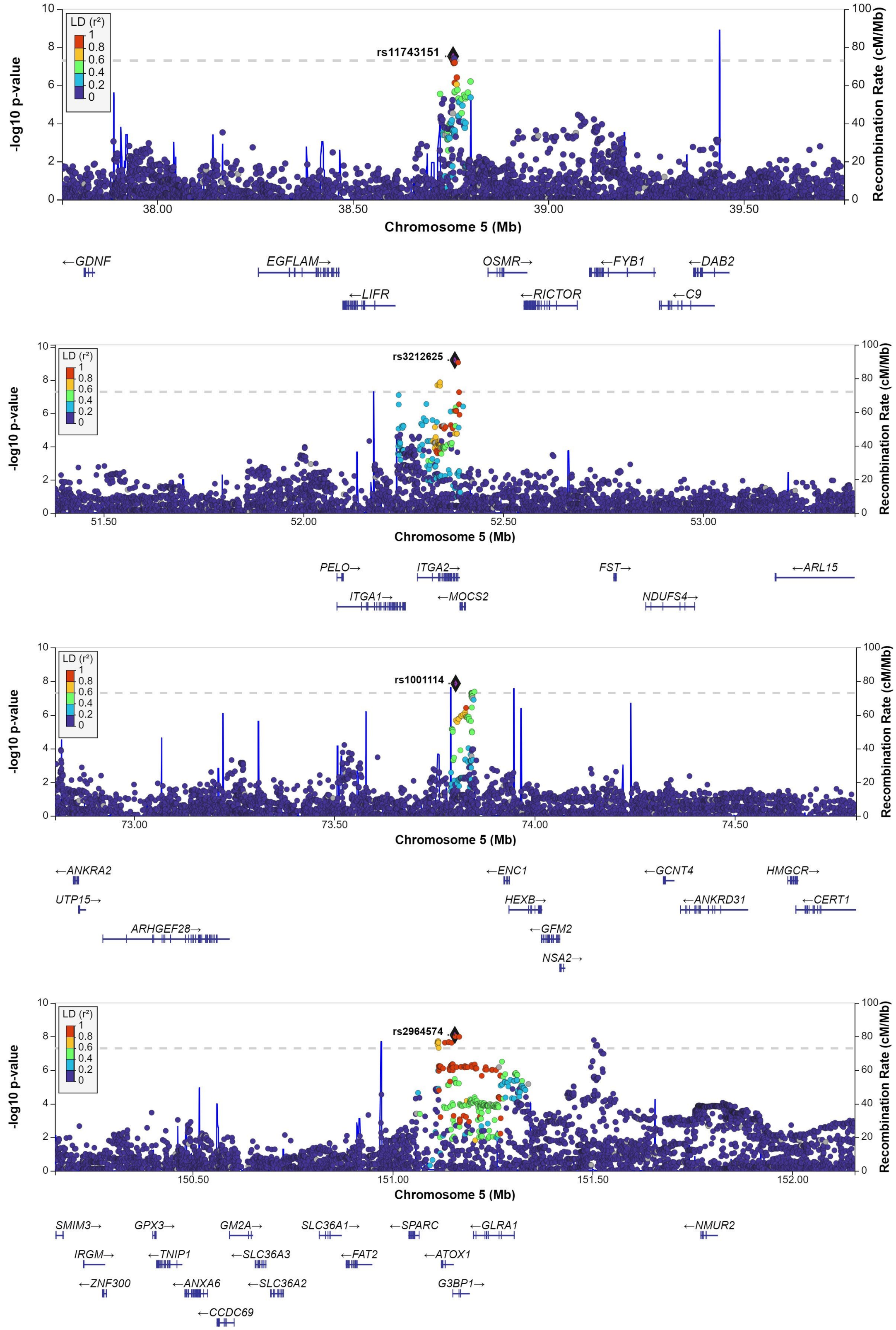

LD (r²)
1
0.8
0.6
0.4
0.2
0
-log10 p-value
Recombination Rate (cM/Mb)
rs11743151
Chromosome 5 (Mb)
38.00
38.50
39.00
39.50
←GDNF
EGFLAM→
←LIFR
OSMR→
←RICTOR
←FYB1
←DAB2
←C9
rs3212625
51.50
52.00
52.50
53.00
PELO→
ITGA2→
ITGA1→
←MOCS2
FST→
NDUFS4→
←ARL15
rs1001114
73.00
73.50
74.00
74.50
←ANKRA2
UTP15→
ARHGEF28→
←ENC1
HEXB→
←GFM2
NSA2→
←GCNT4
←ANKRD31
HMGCR→
←CERT1
rs2964574
150.50
151.00
151.50
152.00
SMIM3→
IRGM→
←ZNF300
GPX3→
←TNIP1
←ANXA6
←CCDC69
GM2A→
←SLC36A3
←SLC36A2
SLC36A1→
←FAT2
←SPARC
←ATOX1
G3BP1→
←GLRA1
←NMUR2

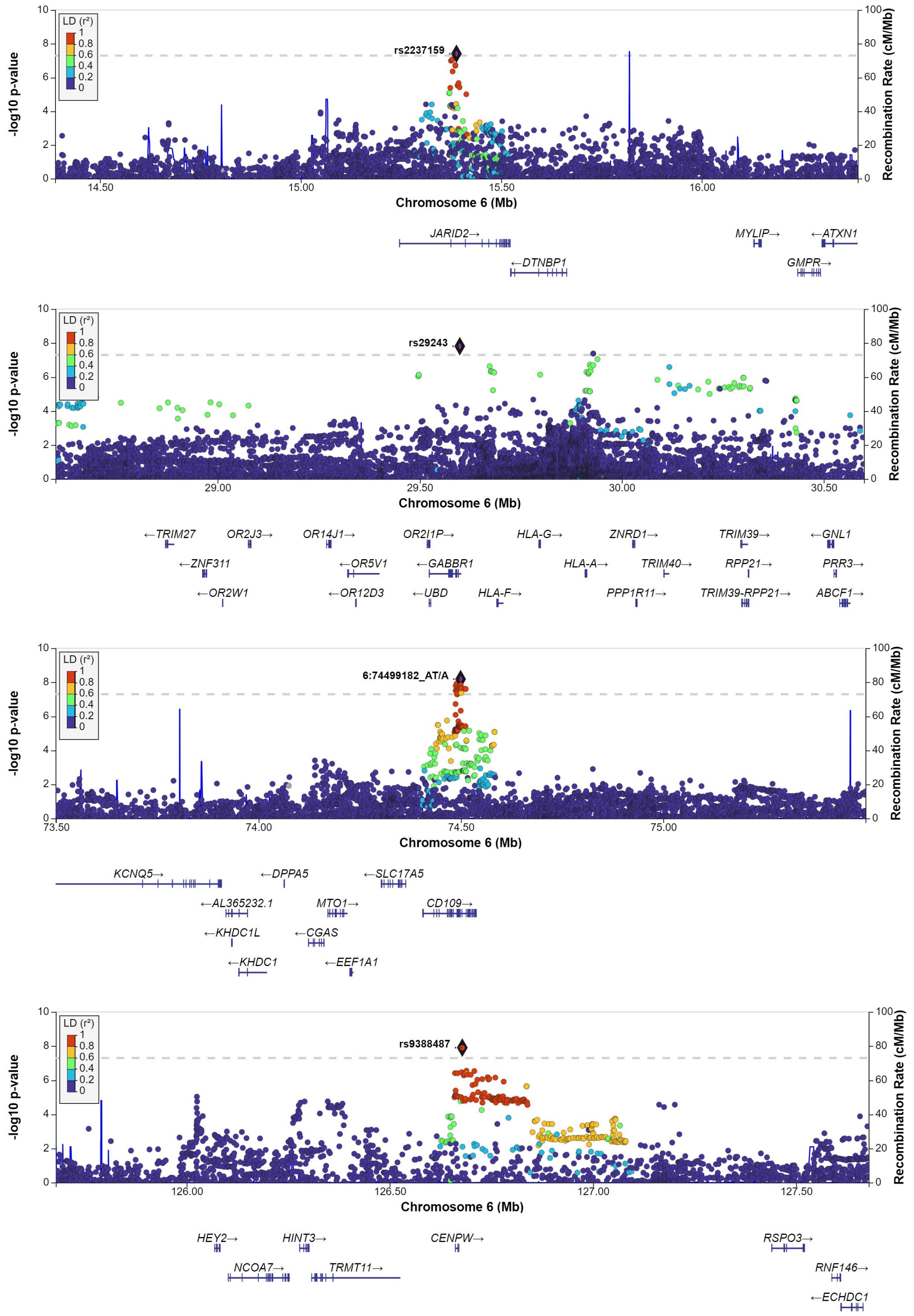

LD (r²)
-log10 p-value
Recombination Rate (cM/Mb)
rs2237159
Chromosome 6 (Mb)
JARID2→
←DTNBP1
MYLIP→
←ATXN1
GMPR→
rs29243
←TRIM27
OR2J3→
OR14J1→
OR2I1P→
HLA-G→
ZNRD1→
TRIM39→
←GNL1
←ZNF311
←OR5V1
←GABBR1
HLA-A→
TRIM40→
RPP21→
PRR3→
←OR2W1
←OR12D3
←UBD
HLA-F→
PPP1R11→
TRIM39-RPP21→
ABCF1→
6:74499182_AT/A
KCNQ5→
←DPPA5
←SLC17A5
←AL365232.1
MTO1→
CD109→
←KHDC1L
←CGAS
←KHDC1
←EEF1A1
rs9388487
HEY2→
HINT3→
CENPW→
RSPO3→
NCOA7→
TRMT11→
RNF146→
←ECHDC1

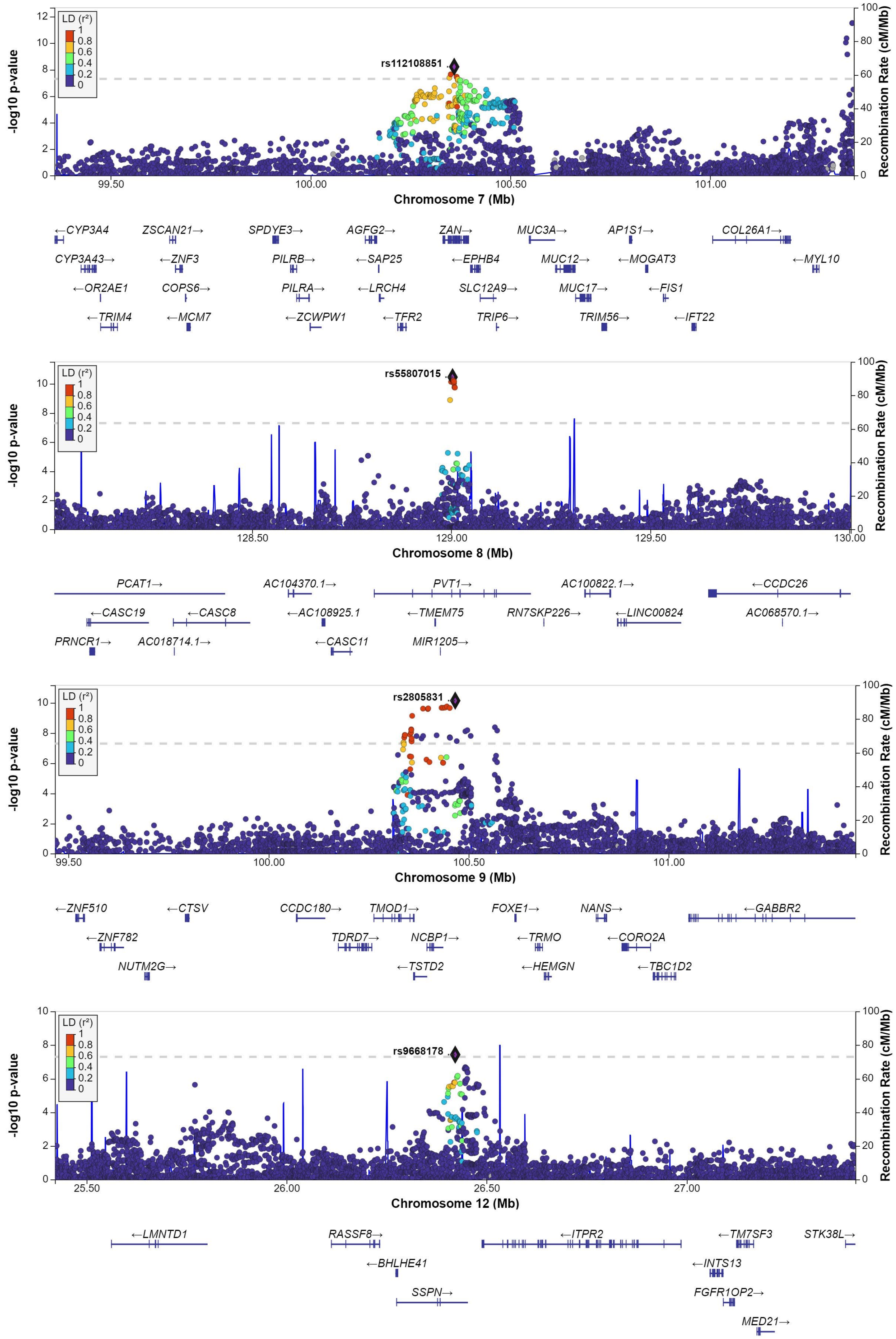

LD (r²)
1
0.8
0.6
0.4
0.2
0
-log10 p-value
Recombination Rate (cM/Mb)
rs112108851
Chromosome 7 (Mb)
99.50
100.00
100.50
101.00
←CYP3A4
ZSCAN21→
SPDYE3→
AGFG2→
ZAN→
MUC3A→
AP1S1→
COL26A1→
CYP3A43→
←ZNF3
PILRB→
←SAP25
←EPHB4
MUC12→
←MOGAT3
←MYL10
←OR2AE1
COPS6→
PILRA→
←LRCH4
SLC12A9→
MUC17→
←FIS1
←TRIM4
←MCM7
←ZCWPW1
←TFR2
TRIP6→
TRIM56→
←IFT22
rs55807015
Chromosome 8 (Mb)
128.50
129.00
129.50
130.00
PCAT1→
AC104370.1→
PVT1→
AC100822.1→
←CCDC26
←CASC19
←CASC8
←AC108925.1
←TMEM75
RN7SKP226→
←LINC00824
AC068570.1→
PRNCR1→
AC018714.1→
←CASC11
MIR1205→
rs2805831
Chromosome 9 (Mb)
99.50
100.00
100.50
101.00
←ZNF510
←CTSV
CCDC180→
TMOD1→
FOXE1→
NANS→
←GABBR2
←ZNF782
TDRD7→
NCBP1→
←TRMO
←CORO2A
NUTM2G→
←TSTD2
←HEMGN
←TBC1D2
rs9668178
Chromosome 12 (Mb)
25.50
26.00
26.50
27.00
←LMNTD1
RASSF8→
←ITPR2
←TM7SF3
STK38L→
←BHLHE41
←INTS13
SSPN→
FGFR1OP2→
MED21→

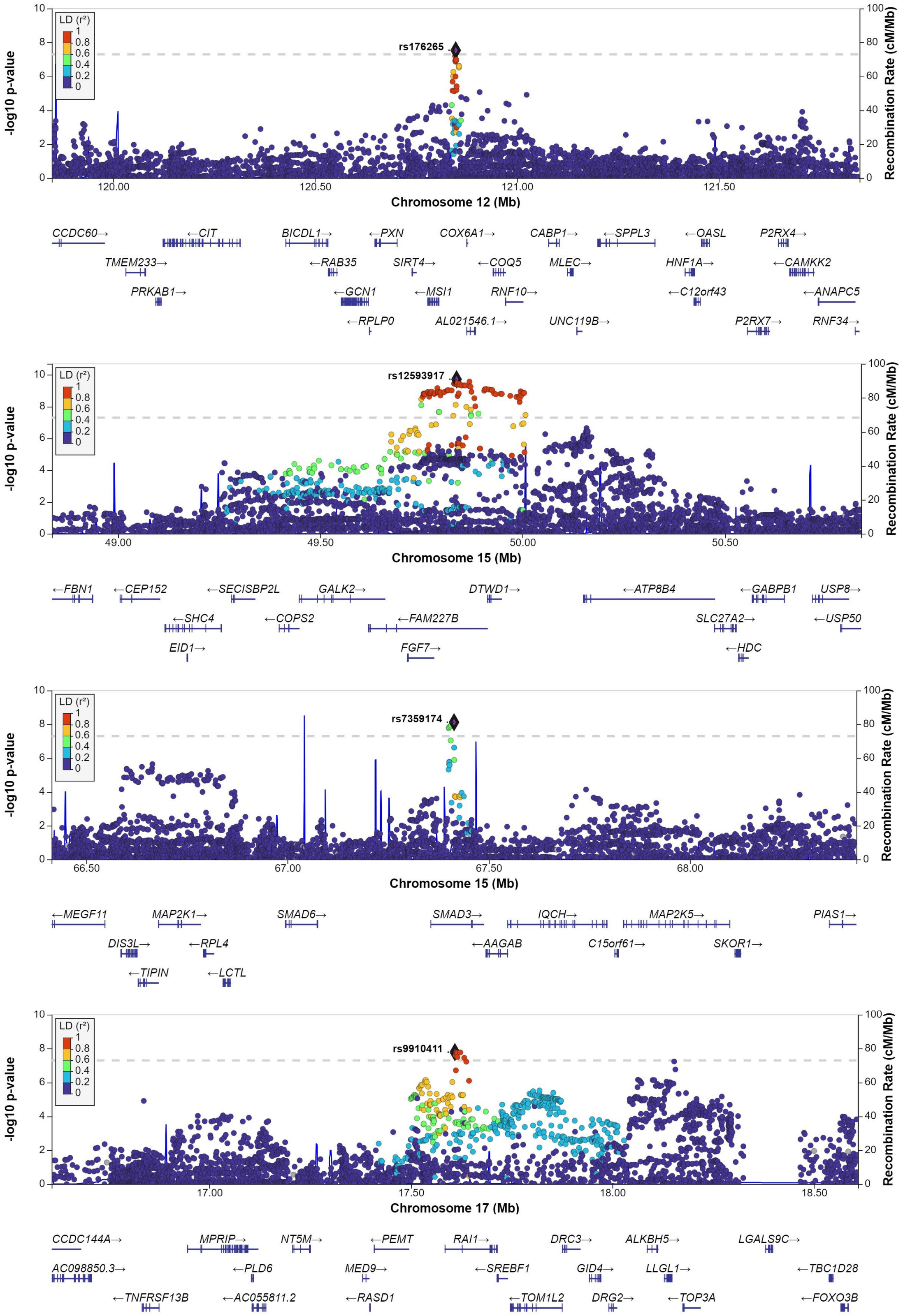

LD (r²)
1
0.8
0.6
0.4
0.2
0
rs176265
-log10 p-value
Recombination Rate (cM/Mb)
Chromosome 12 (Mb)
CCDC60→
←CIT
BICDL1→
←PXN
COX6A1→
CABP1→
←SPPL3
←OASL
P2RX4→
TMEM233→
←RAB35
SIRT4→
←COQ5
MLEC→
HNF1A→
←CAMKK2
PRKAB1→
←GCN1
←MSI1
RNF10→
←C12orf43
←ANAPC5
←RPLP0
AL021546.1→
UNC119B→
P2RX7→
RNF34→
rs12593917
Chromosome 15 (Mb)
←FBN1
←CEP152
←SECISBP2L
GALK2→
DTWD1→
←ATP8B4
←GABPB1
USP8→
←SHC4
←COPS2
←FAM227B
SLC27A2→
←USP50
EID1→
FGF7→
←HDC
rs7359174
Chromosome 15 (Mb)
←MEGF11
MAP2K1→
SMAD6→
SMAD3→
IQCH→
MAP2K5→
PIAS1→
DIS3L→
←RPL4
←AAGAB
C15orf61→
SKOR1→
←TIPIN
←LCTL
rs9910411
Chromosome 17 (Mb)
CCDC144A→
MPRIP→
NT5M→
←PEMT
RAI1→
DRC3→
ALKBH5→
LGALS9C→
AC098850.3→
←PLD6
MED9→
←SREBF1
GID4→
LLGL1→
←TBC1D28
←TNFRSF13B
←AC055811.2
←RASD1
←TOM1L2
DRG2→
←TOP3A
←FOXO3B

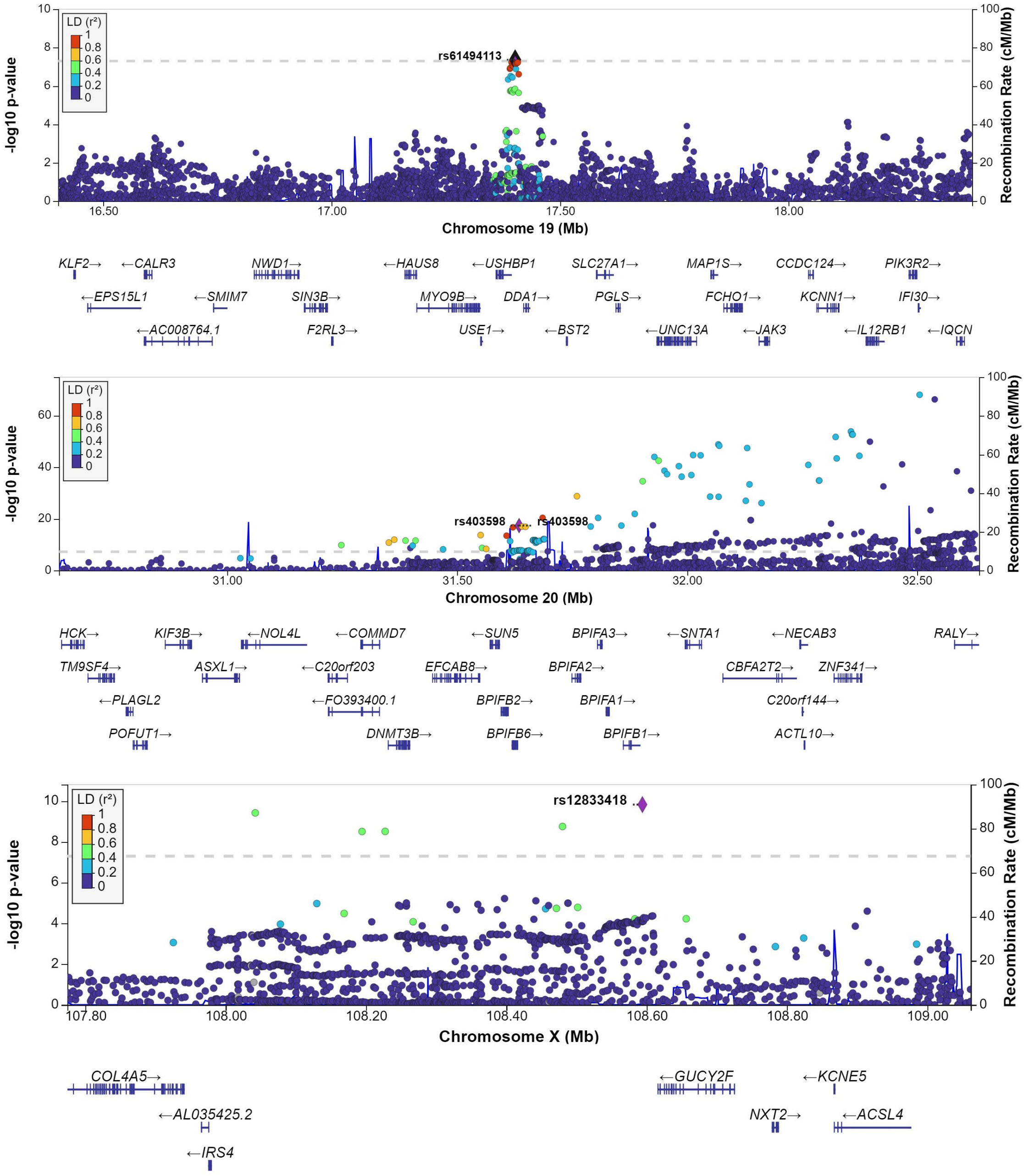

LD (r²)
1
0.8
0.6
0.4
0.2
0
-log10 p-value
Recombination Rate (cM/Mb)
rs61494113
Chromosome 19 (Mb)
16.50
17.00
17.50
18.00
KLF2→
←CALR3
NWD1→
←HAUS8
←USHBP1
SLC27A1→
MAP1S→
CCDC124→
PIK3R2→
←EPS15L1
←SMIM7
SIN3B→
MYO9B→
DDA1→
PGLS→
FCHO1→
KCNN1→
IFI30→
←AC008764.1
F2RL3→
USE1→
←BST2
←UNC13A
←JAK3
←IL12RB1
←IQCN
rs403598
rs403598
Chromosome 20 (Mb)
31.00
31.50
32.00
32.50
HCK→
KIF3B→
←NOL4L
←COMMD7
←SUN5
BPIFA3→
←SNTA1
←NECAB3
RALY→
TM9SF4→
ASXL1→
←C20orf203
EFCAB8→
BPIFA2→
CBFA2T2→
ZNF341→
←PLAGL2
←FO393400.1
BPIFB2→
BPIFA1→
C20orf144→
POFUT1→
DNMT3B→
BPIFB6→
BPIFB1→
ACTL10→
rs12833418
Chromosome X (Mb)
107.80
108.00
108.20
108.40
108.60
108.80
109.00
COL4A5→
←GUCY2F
←KCNE5
←AL035425.2
NXT2→
←ACSL4
←IRS4

Supplementary Figure 4. Forest plots of new BCC loci identified in the European ancestry GWA meta-analysis. The error bars correspond to the 95% CIs.

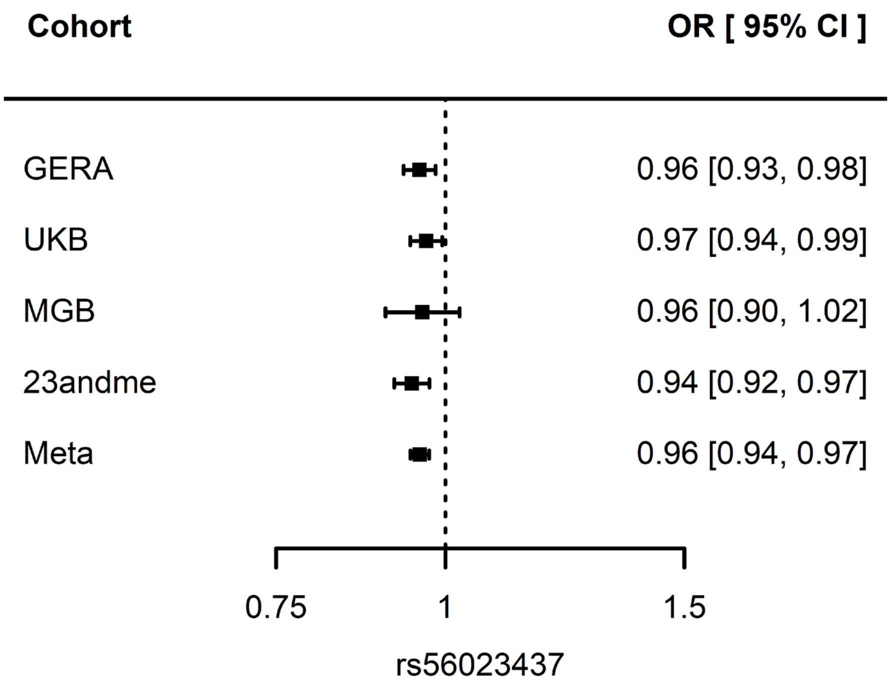

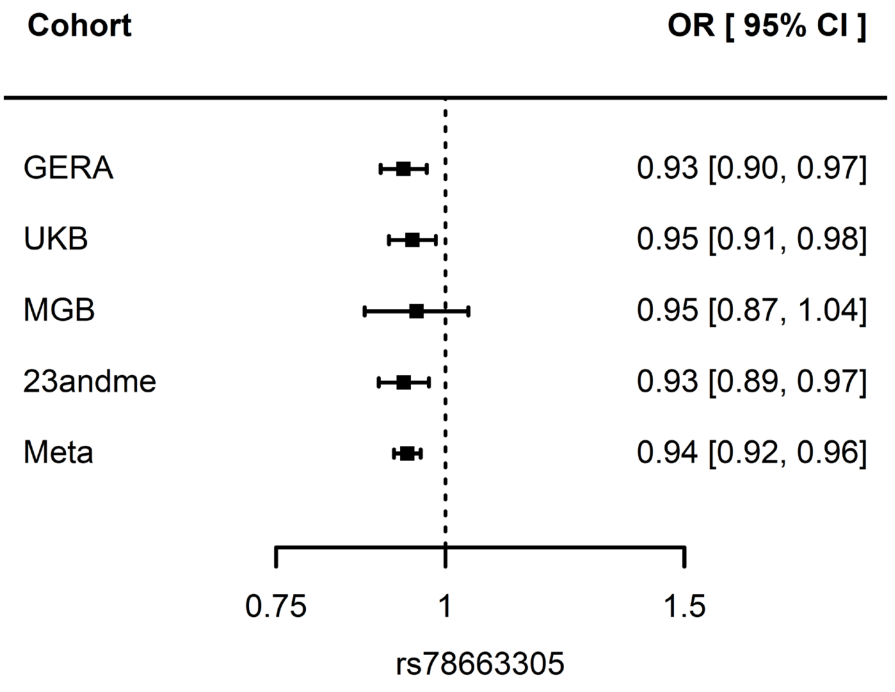

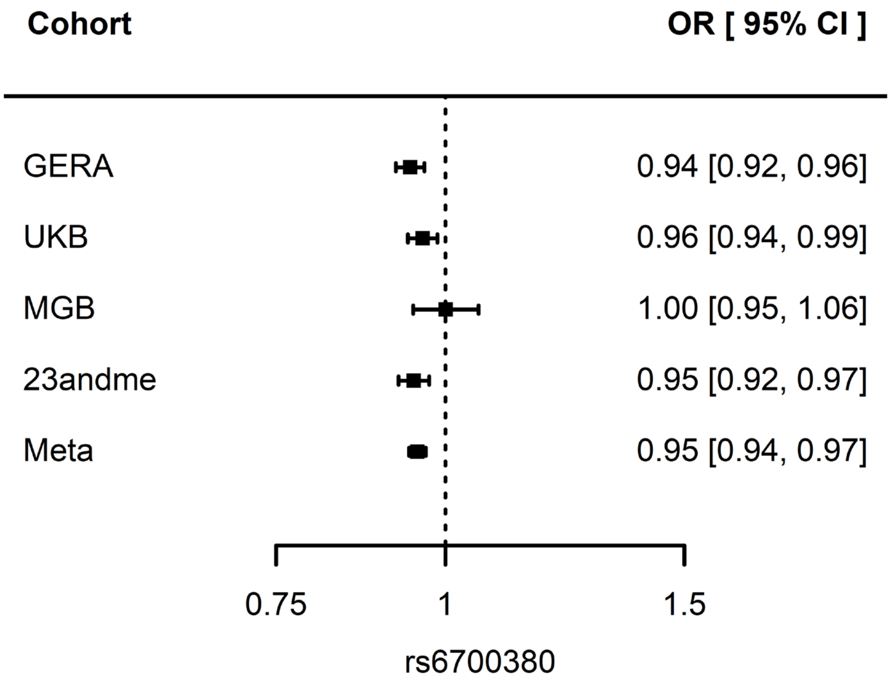
Cohort
OR [95% CI]
GERA
0.94 [0.92, 0.96]
UKB
0.96 [0.94, 0.99]
MGB
1.00 [0.95, 1.06]
23andme
0.95 [0.92, 0.97]
Meta
0.95 [0.94, 0.97]
0.75
1
1.5
rs6700380

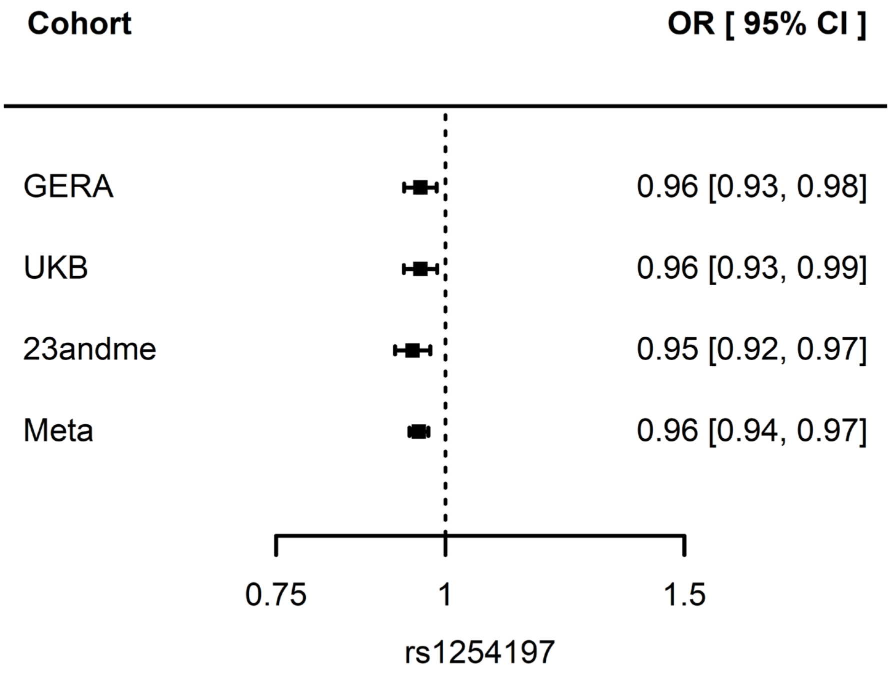
Cohort
OR [95% CI]
GERA
0.96 [0.93, 0.98]
UKB
0.96 [0.93, 0.99]
23andme
0.95 [0.92, 0.97]
Meta
0.96 [0.94, 0.97]
0.75
1
1.5
rs1254197

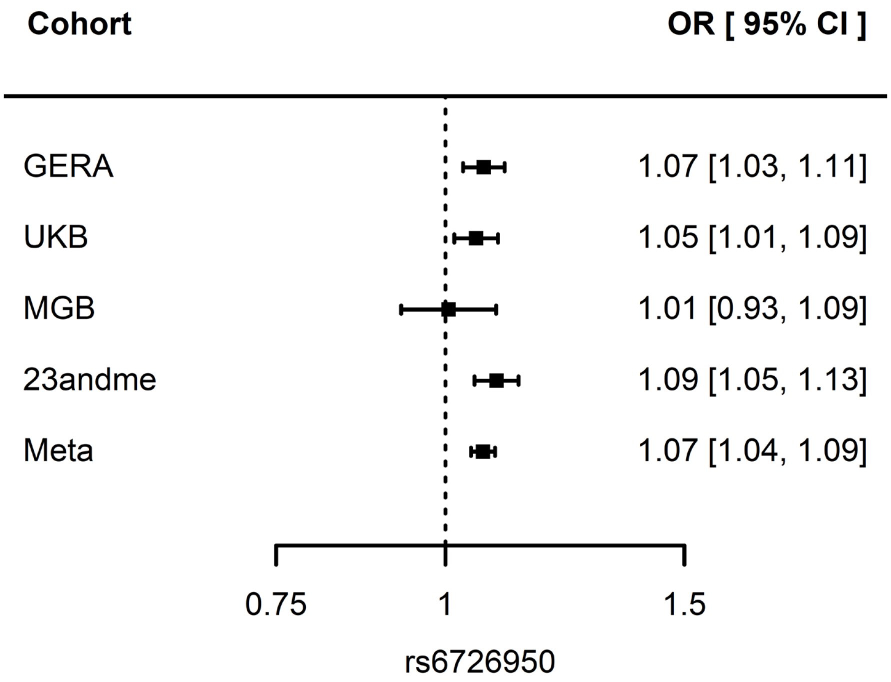
Cohort
OR [95% CI]
GERA
1.07 [1.03, 1.11]
UKB
1.05 [1.01, 1.09]
MGB
1.01 [0.93, 1.09]
23andme
1.09 [1.05, 1.13]
Meta
1.07 [1.04, 1.09]
0.75
1
1.5
rs6726950

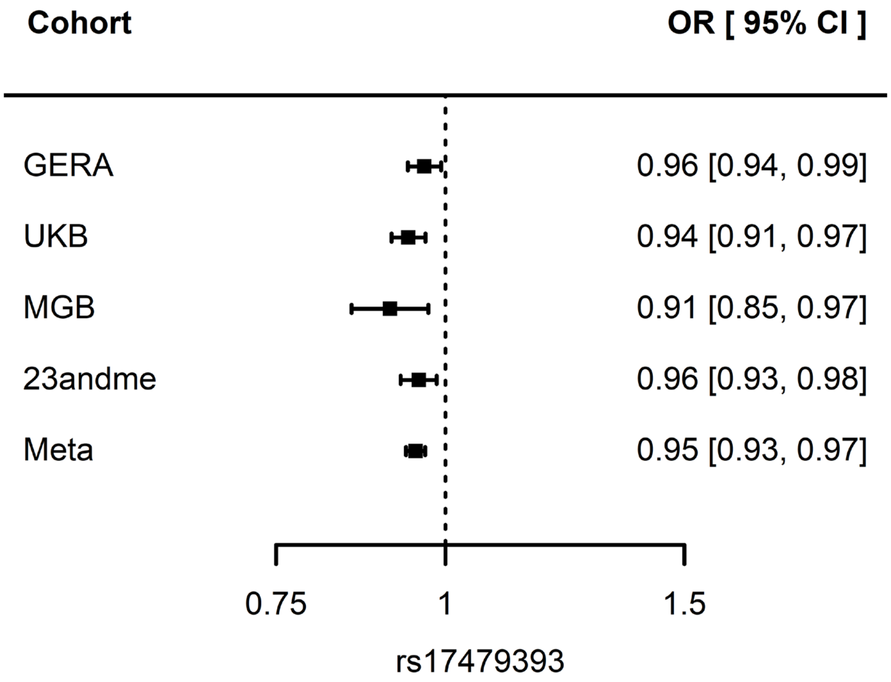
Cohort
OR [95% CI]
GERA
0.96 [0.94, 0.99]
UKB
0.94 [0.91, 0.97]
MGB
0.91 [0.85, 0.97]
23andme
0.96 [0.93, 0.98]
Meta
0.95 [0.93, 0.97]
0.75
1
1.5
rs17479393

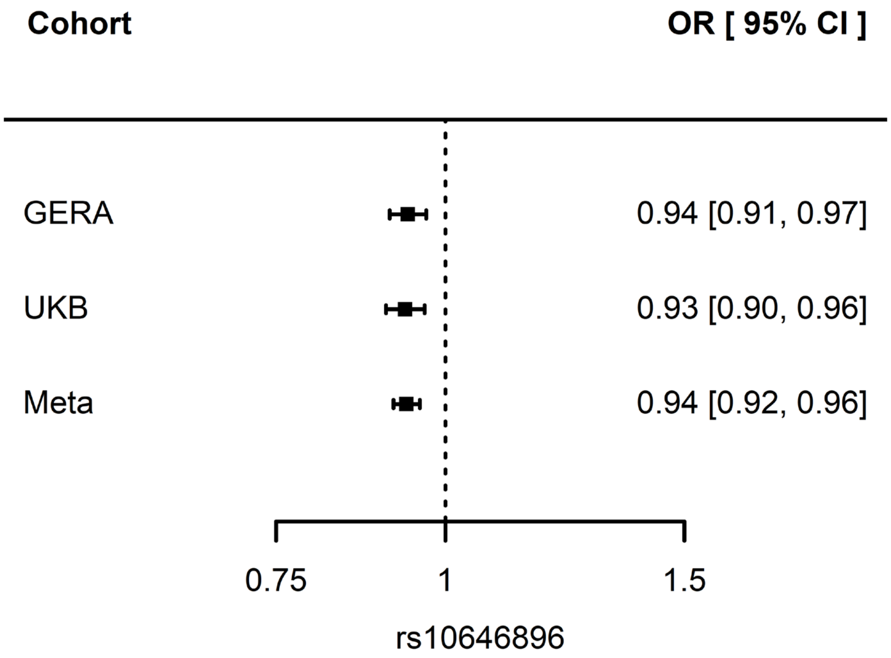
Cohort
OR [95% CI]
GERA
0.94 [0.91, 0.97]
UKB
0.93 [0.90, 0.96]
Meta
0.94 [0.92, 0.96]
0.75
1
1.5
rs10646896

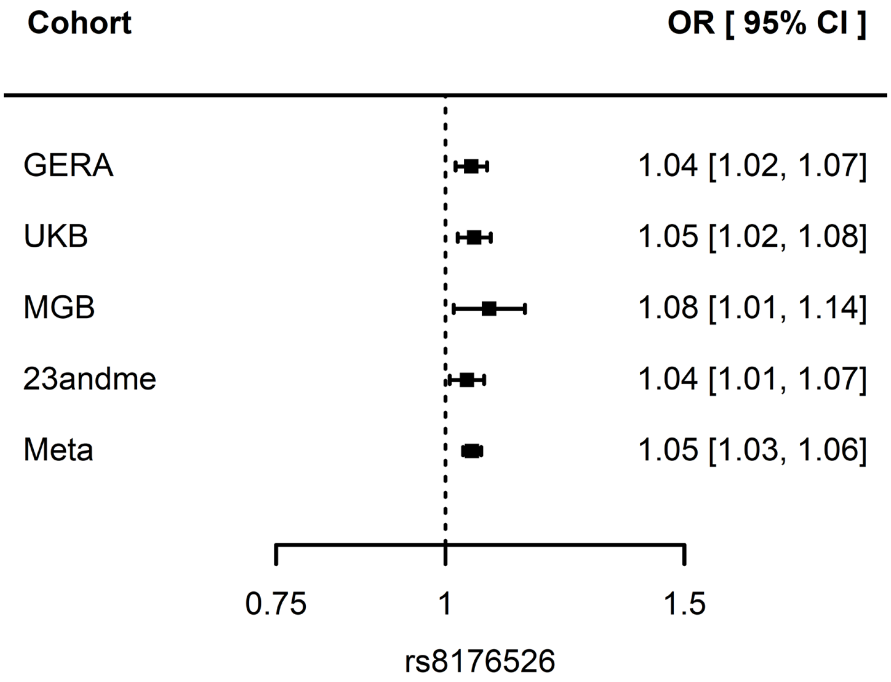
Cohort
OR [95% CI]
GERA
1.04 [1.02, 1.07]
UKB
1.05 [1.02, 1.08]
MGB
1.08 [1.01, 1.14]
23andme
1.04 [1.01, 1.07]
Meta
1.05 [1.03, 1.06]
0.75
1
1.5
rs8176526

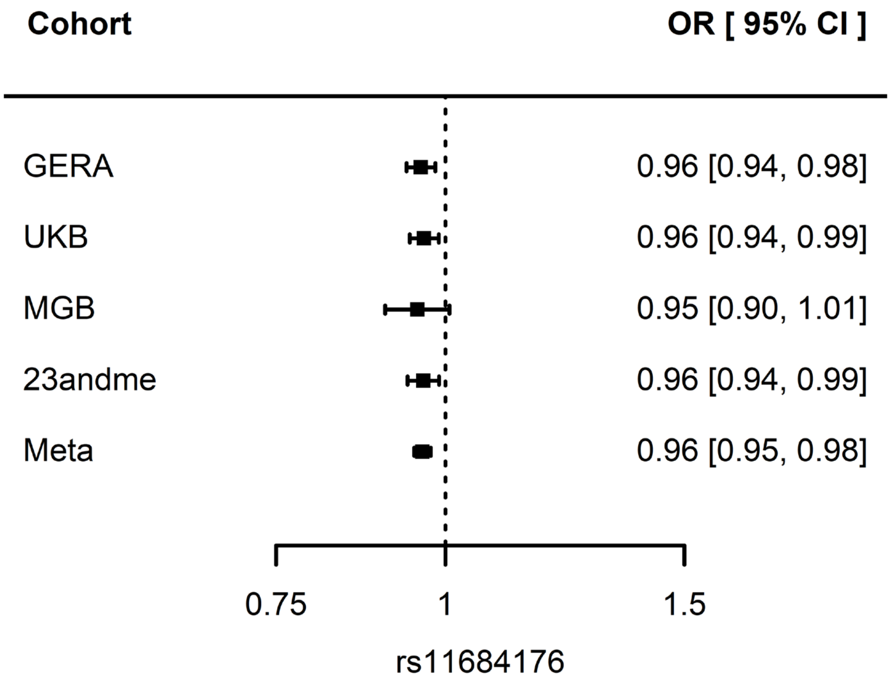
Cohort
OR [95% CI]
GERA
0.96 [0.94, 0.98]
UKB
0.96 [0.94, 0.99]
MGB
0.95 [0.90, 1.01]
23andme
0.96 [0.94, 0.99]
Meta
0.96 [0.95, 0.98]
0.75
1
1.5
rs11684176

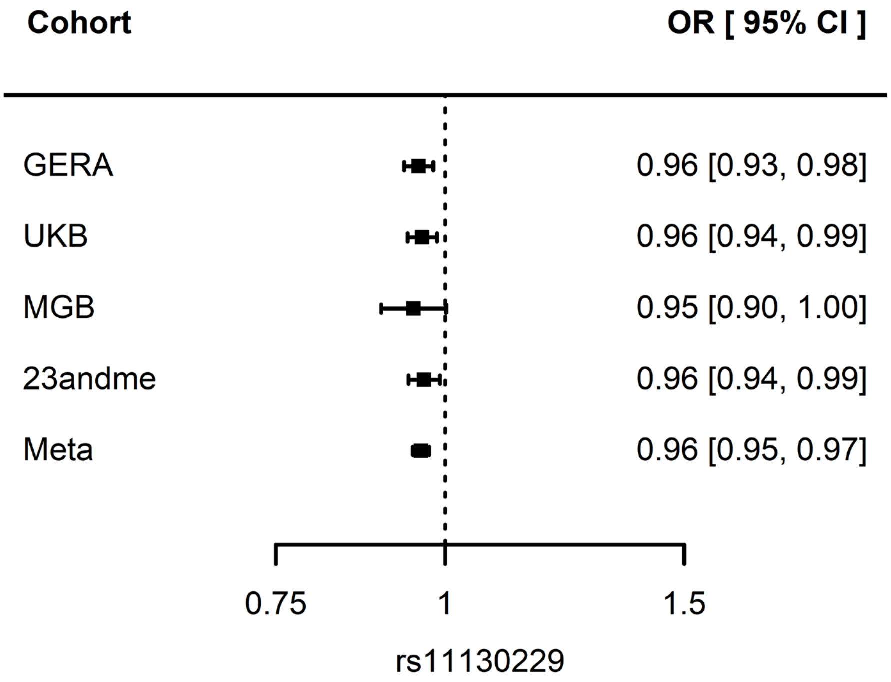
Cohort
OR [95% CI]
GERA
0.96 [0.93, 0.98]
UKB
0.96 [0.94, 0.99]
MGB
0.95 [0.90, 1.00]
23andme
0.96 [0.94, 0.99]
Meta
0.96 [0.95, 0.97]
0.75
1
1.5
rs11130229

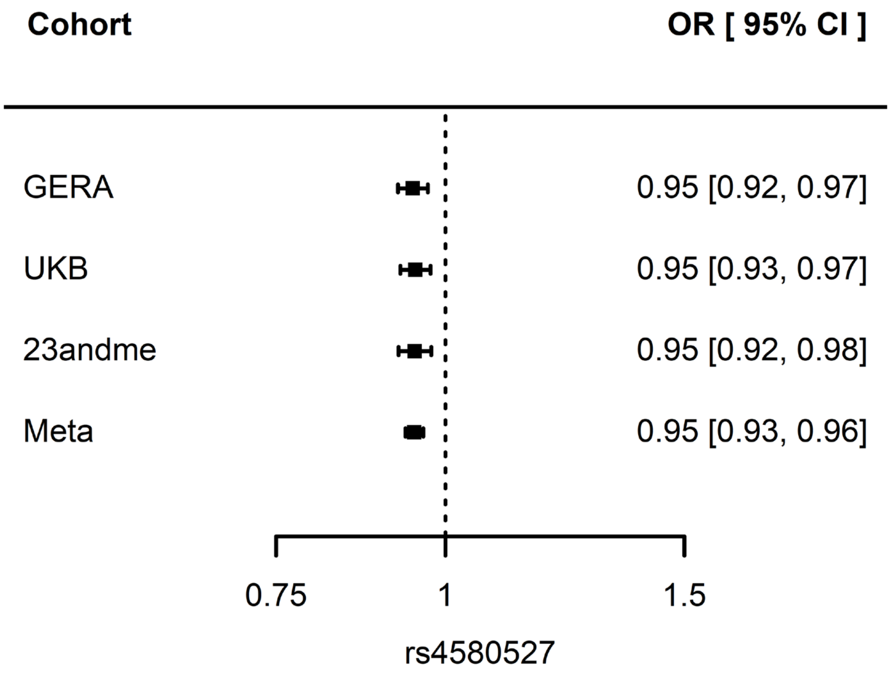
Cohort
OR [95% CI]
GERA
0.95 [0.92, 0.97]
UKB
0.95 [0.93, 0.97]
23andme
0.95 [0.92, 0.98]
Meta
0.95 [0.93, 0.96]
0.75
1
1.5
rs4580527

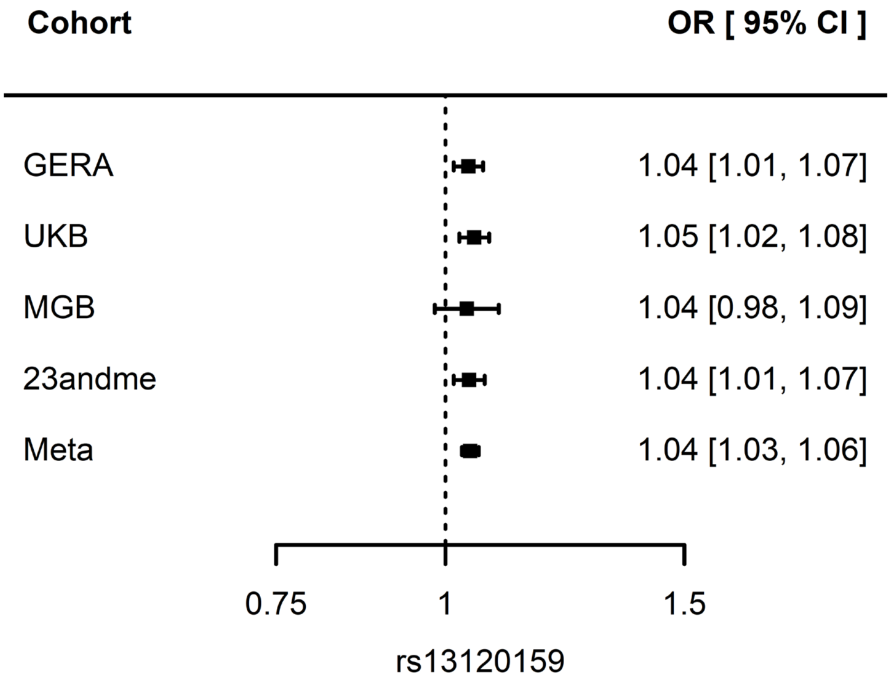
Cohort
OR [95% CI]
GERA
1.04 [1.01, 1.07]
UKB
1.05 [1.02, 1.08]
MGB
1.04 [0.98, 1.09]
23andme
1.04 [1.01, 1.07]
Meta
1.04 [1.03, 1.06]
0.75
1
1.5
rs13120159

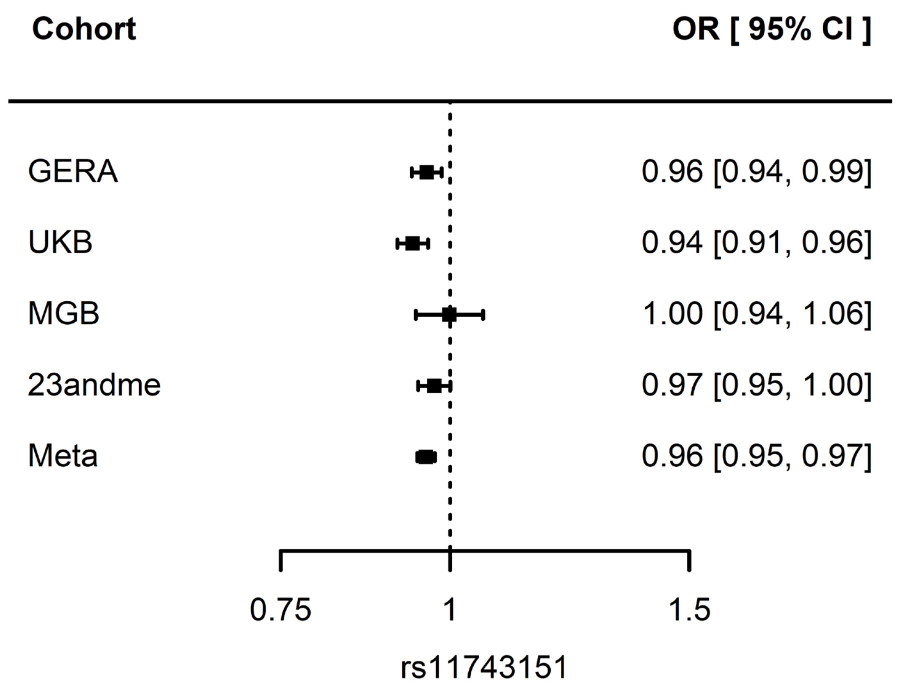
Cohort
OR [95% CI]
GERA
0.96 [0.94, 0.99]
UKB
0.94 [0.91, 0.96]
MGB
1.00 [0.94, 1.06]
23andme
0.97 [0.95, 1.00]
Meta
0.96 [0.95, 0.97]
0.75
1
1.5
rs11743151

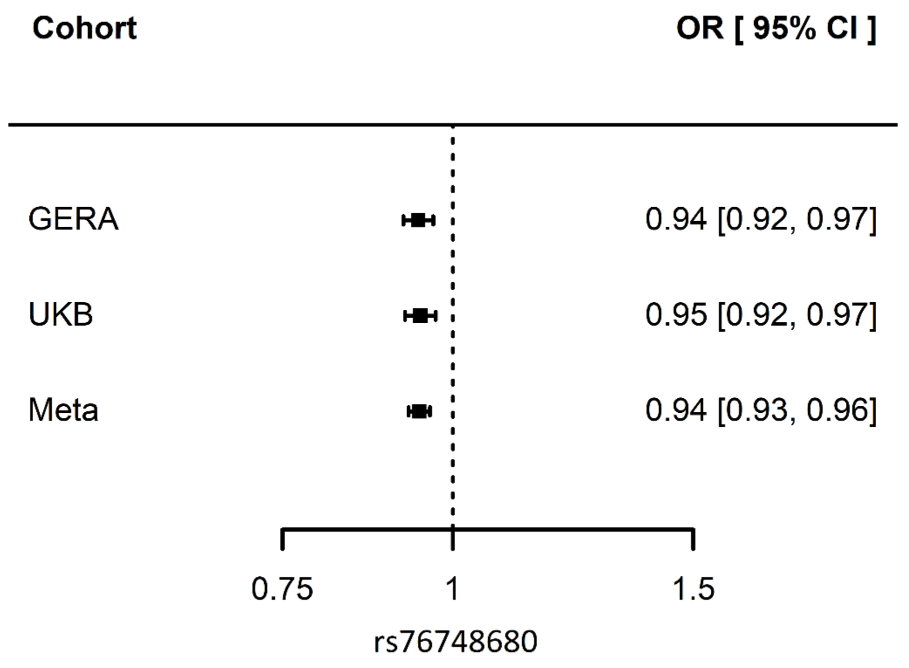
Cohort
OR [95% CI]
GERA
0.94 [0.92, 0.97]
UKB
0.95 [0.92, 0.97]
Meta
0.94 [0.93, 0.96]
0.75
1
1.5
rs76748680

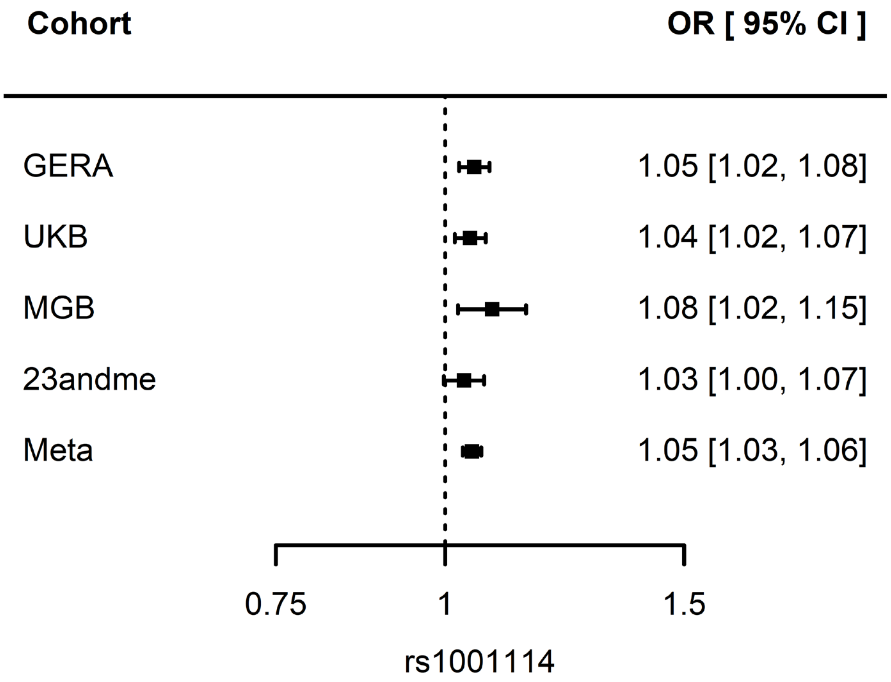
Cohort
OR [95% CI]
GERA
1.05 [1.02, 1.08]
UKB
1.04 [1.02, 1.07]
MGB
1.08 [1.02, 1.15]
23andme
1.03 [1.00, 1.07]
Meta
1.05 [1.03, 1.06]
0.75
1
1.5
rs1001114

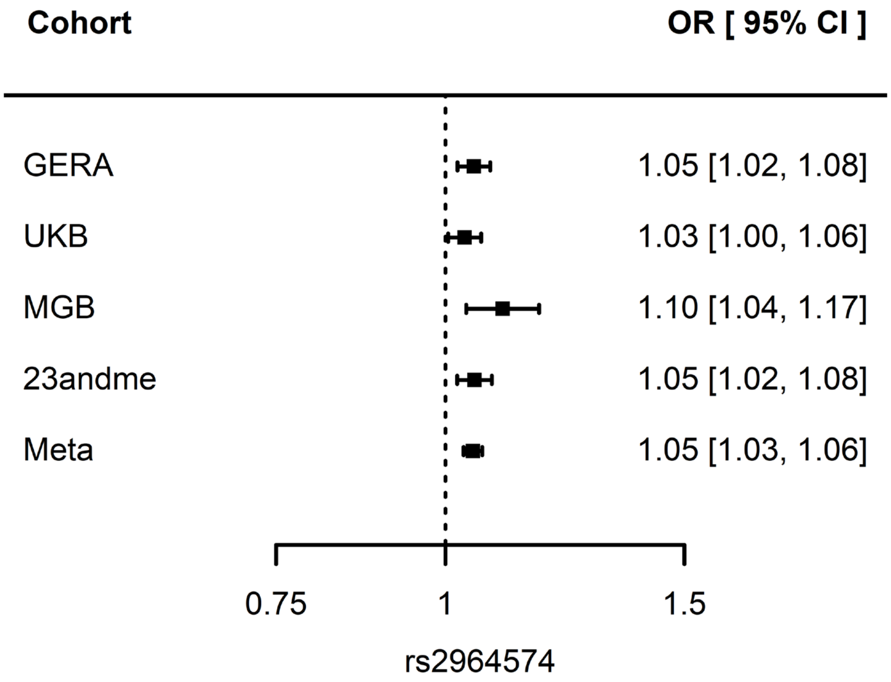
Cohort
OR [95% CI]
GERA
1.05 [1.02, 1.08]
UKB
1.03 [1.00, 1.06]
MGB
1.10 [1.04, 1.17]
23andme
1.05 [1.02, 1.08]
Meta
1.05 [1.03, 1.06]
0.75
1
1.5
rs2964574

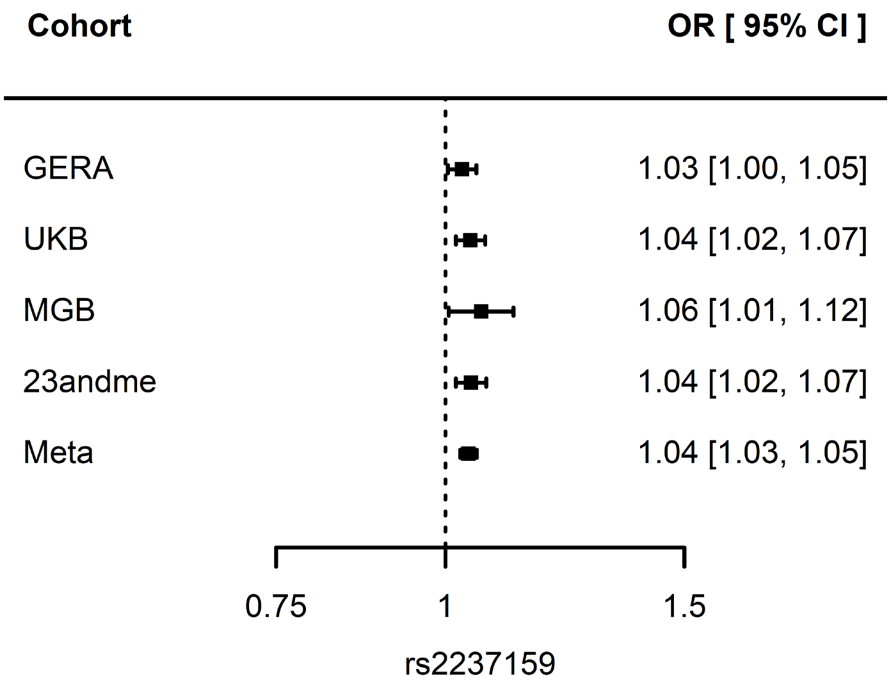
Cohort
OR [95% CI]
GERA
1.03 [1.00, 1.05]
UKB
1.04 [1.02, 1.07]
MGB
1.06 [1.01, 1.12]
23andme
1.04 [1.02, 1.07]
Meta
1.04 [1.03, 1.05]
0.75
1
1.5
rs2237159

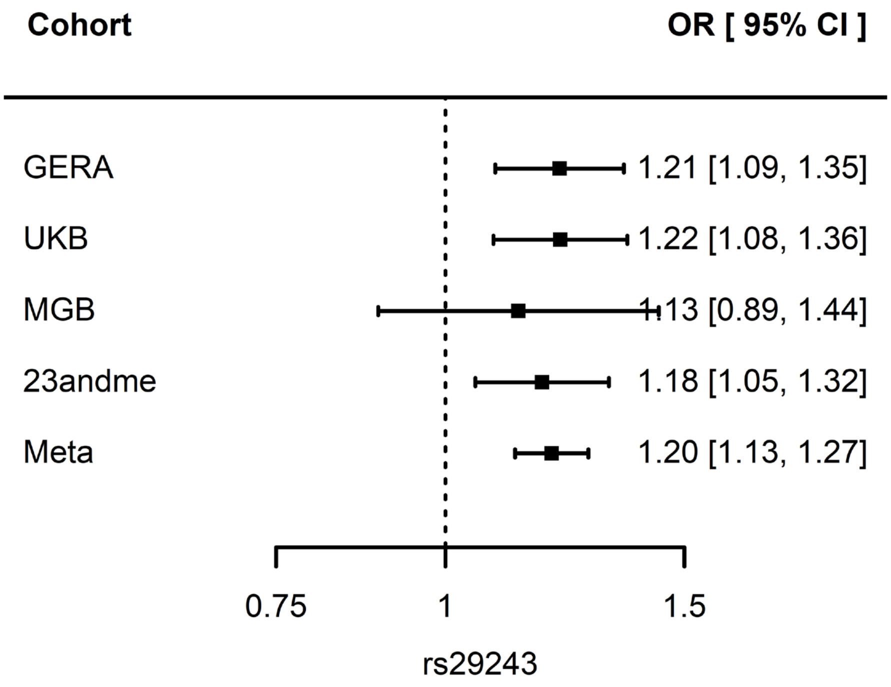
Cohort
OR [95% CI]
GERA
1.21 [1.09, 1.35]
UKB
1.22 [1.08, 1.36]
MGB
1.13 [0.89, 1.44]
23andme
1.18 [1.05, 1.32]
Meta
1.20 [1.13, 1.27]
0.75
1
1.5
rs29243

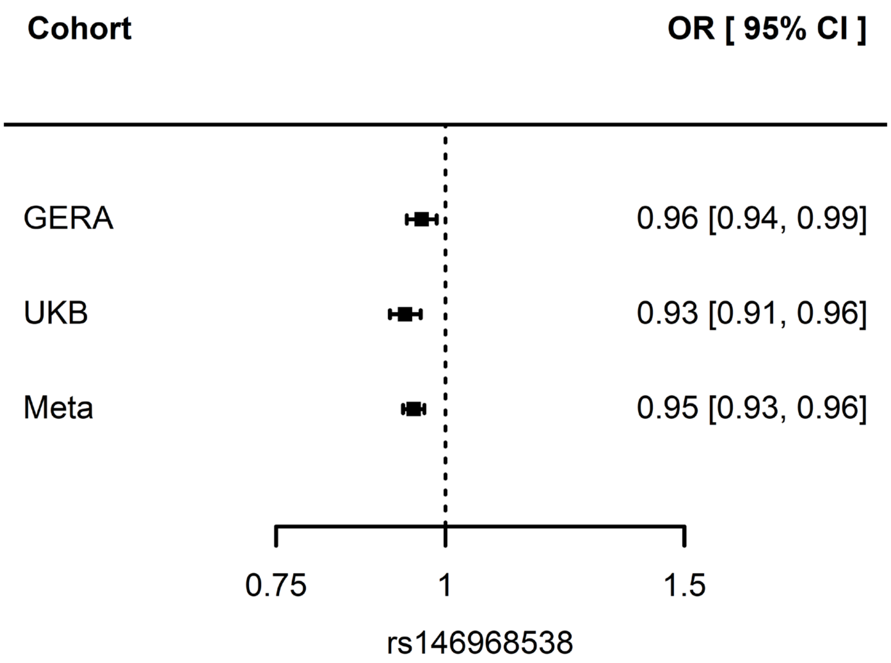
Cohort
OR [95% CI]
GERA
0.96 [0.94, 0.99]
UKB
0.93 [0.91, 0.96]
Meta
0.95 [0.93, 0.96]
0.75
1
1.5
rs146968538

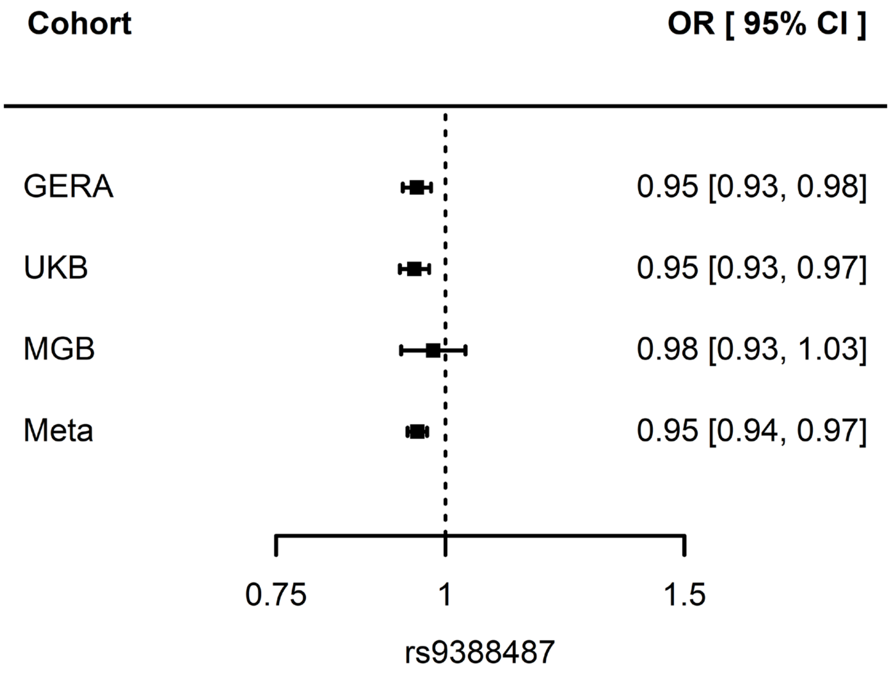
Cohort
OR [95% CI]
GERA
0.95 [0.93, 0.98]
UKB
0.95 [0.93, 0.97]
MGB
0.98 [0.93, 1.03]
Meta
0.95 [0.94, 0.97]
0.75
1
1.5
rs9388487

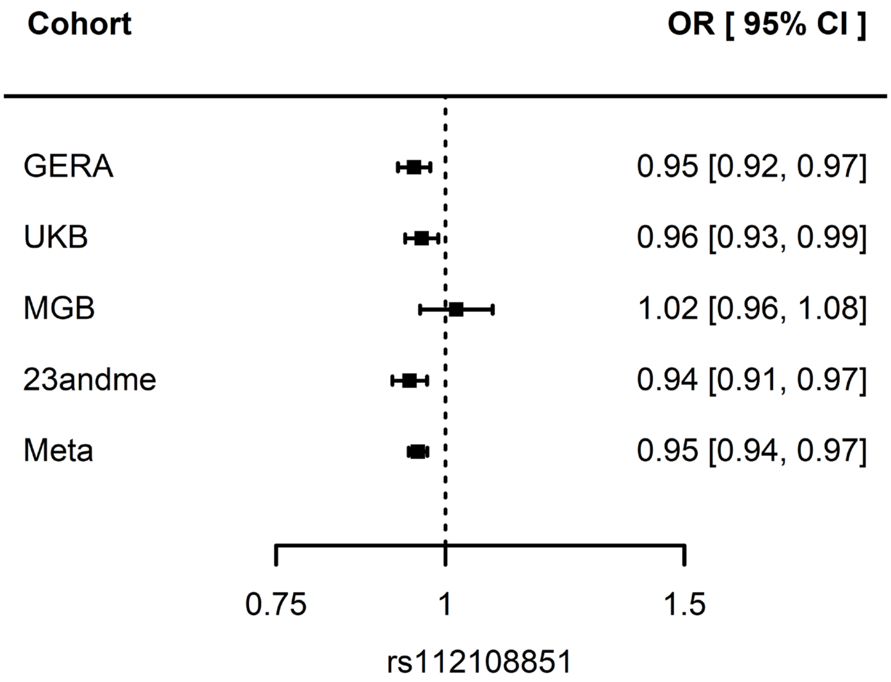
Cohort
OR [95% CI]
GERA
0.95 [0.92, 0.97]
UKB
0.96 [0.93, 0.99]
MGB
1.02 [0.96, 1.08]
23andme
0.94 [0.91, 0.97]
Meta
0.95 [0.94, 0.97]
0.75
1
1.5
rs112108851

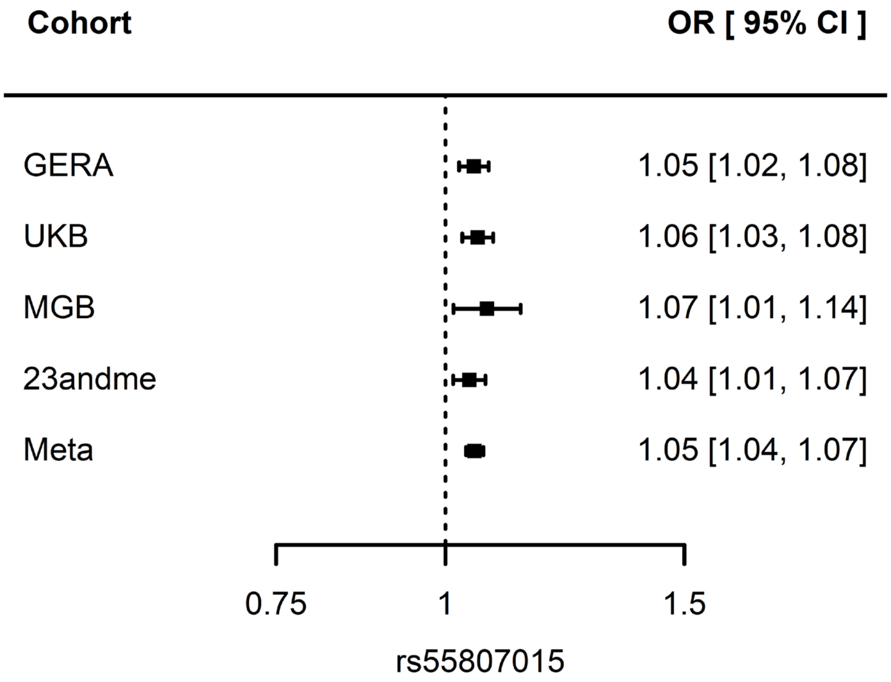
Cohort
OR [95% CI]
GERA
1.05 [1.02, 1.08]
UKB
1.06 [1.03, 1.08]
MGB
1.07 [1.01, 1.14]
23andme
1.04 [1.01, 1.07]
Meta
1.05 [1.04, 1.07]
0.75
1
1.5
rs55807015

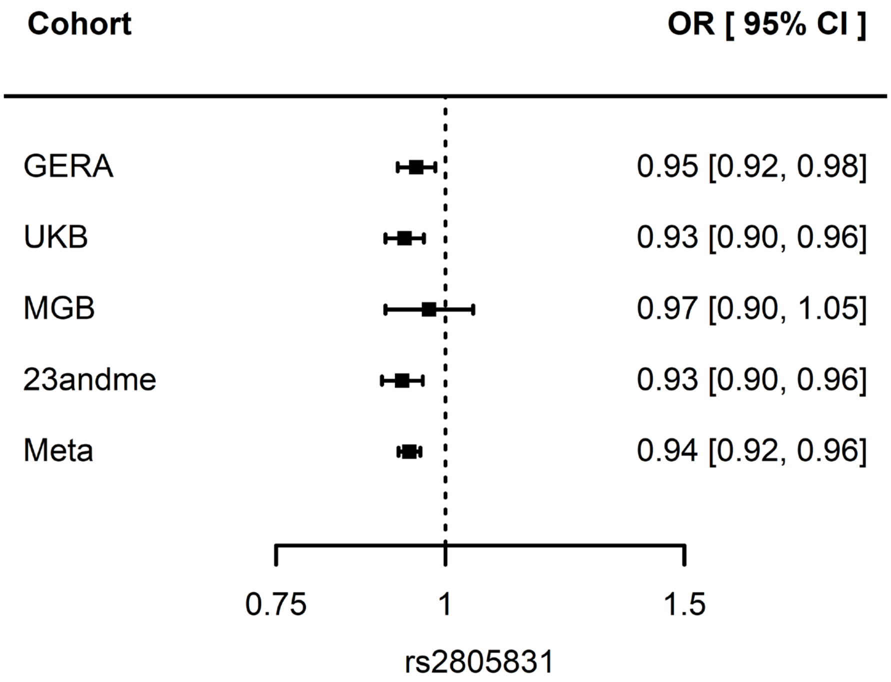
Cohort
OR [95% CI]
GERA
0.95 [0.92, 0.98]
UKB
0.93 [0.90, 0.96]
MGB
0.97 [0.90, 1.05]
23andme
0.93 [0.90, 0.96]
Meta
0.94 [0.92, 0.96]
0.75
1
1.5
rs2805831

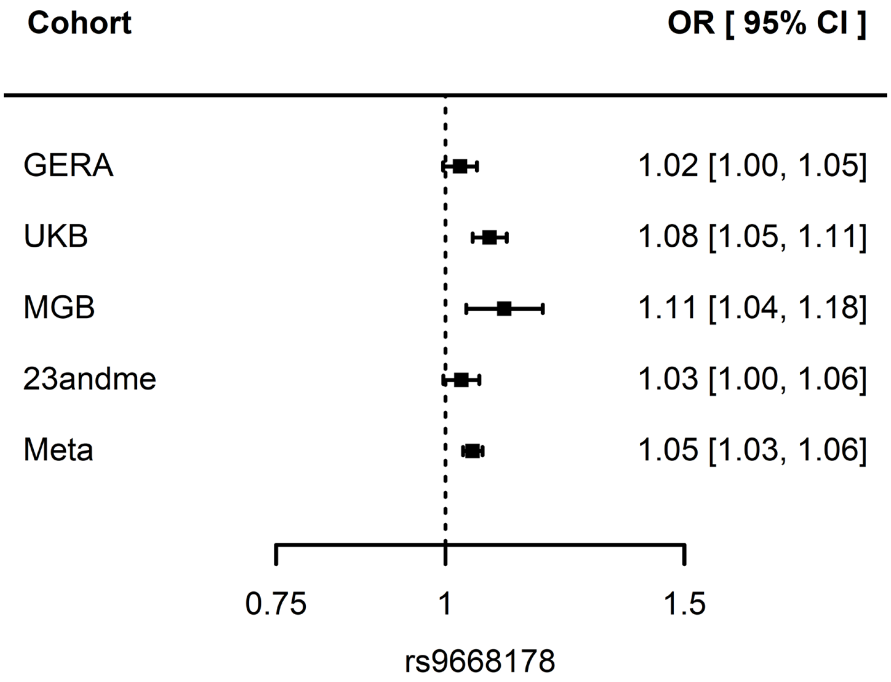
Cohort
OR [95% CI]
GERA
1.02 [1.00, 1.05]
UKB
1.08 [1.05, 1.11]
MGB
1.11 [1.04, 1.18]
23andme
1.03 [1.00, 1.06]
Meta
1.05 [1.03, 1.06]
0.75
1
1.5
rs9668178

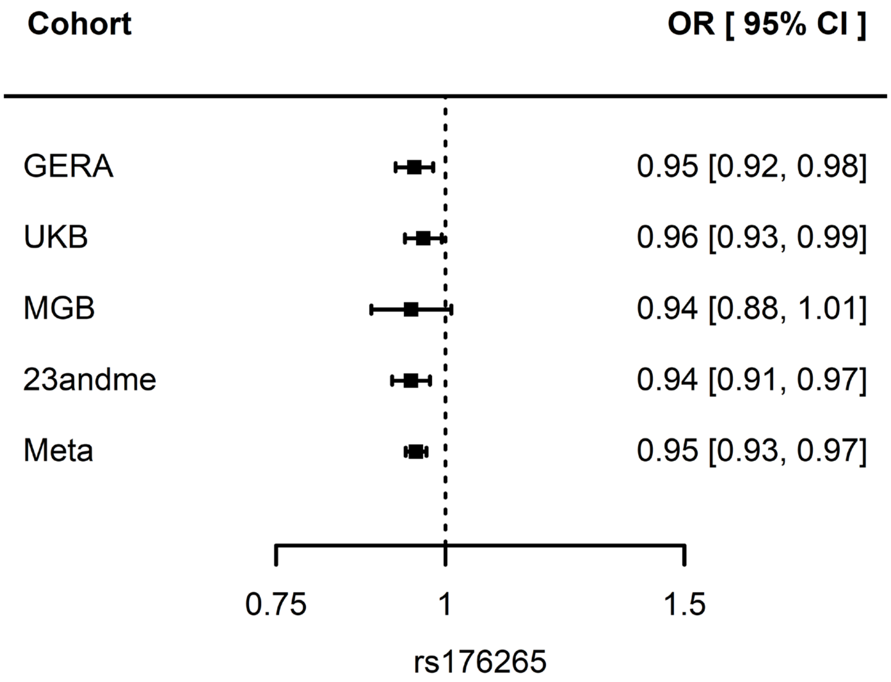
Cohort
OR [95% CI]
GERA
0.95 [0.92, 0.98]
UKB
0.96 [0.93, 0.99]
MGB
0.94 [0.88, 1.01]
23andme
0.94 [0.91, 0.97]
Meta
0.95 [0.93, 0.97]
0.75
1
1.5
rs176265

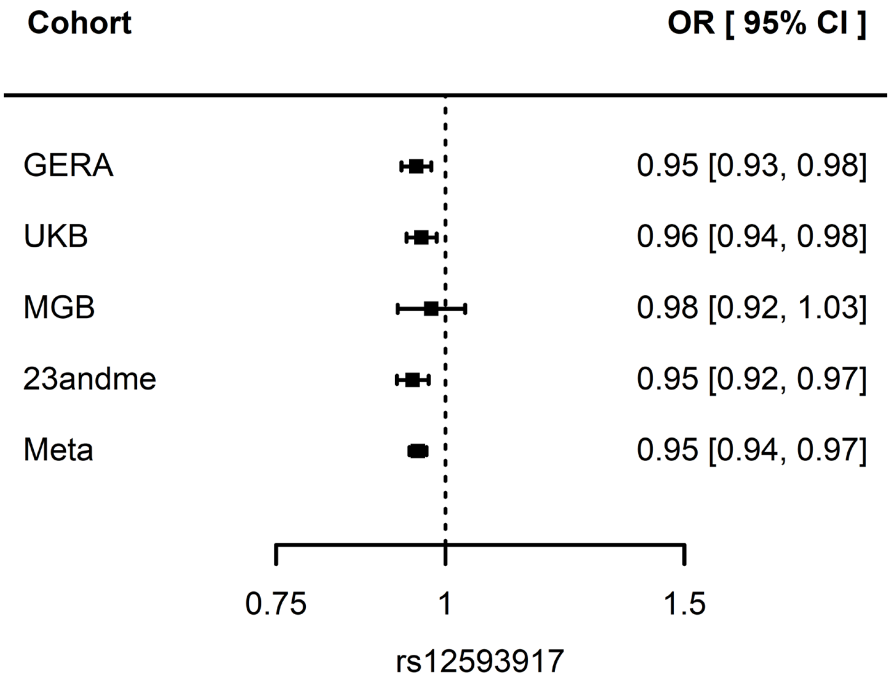
Cohort
OR [95% CI]
GERA
0.95 [0.93, 0.98]
UKB
0.96 [0.94, 0.98]
MGB
0.98 [0.92, 1.03]
23andme
0.95 [0.92, 0.97]
Meta
0.95 [0.94, 0.97]
0.75
1
1.5
rs12593917

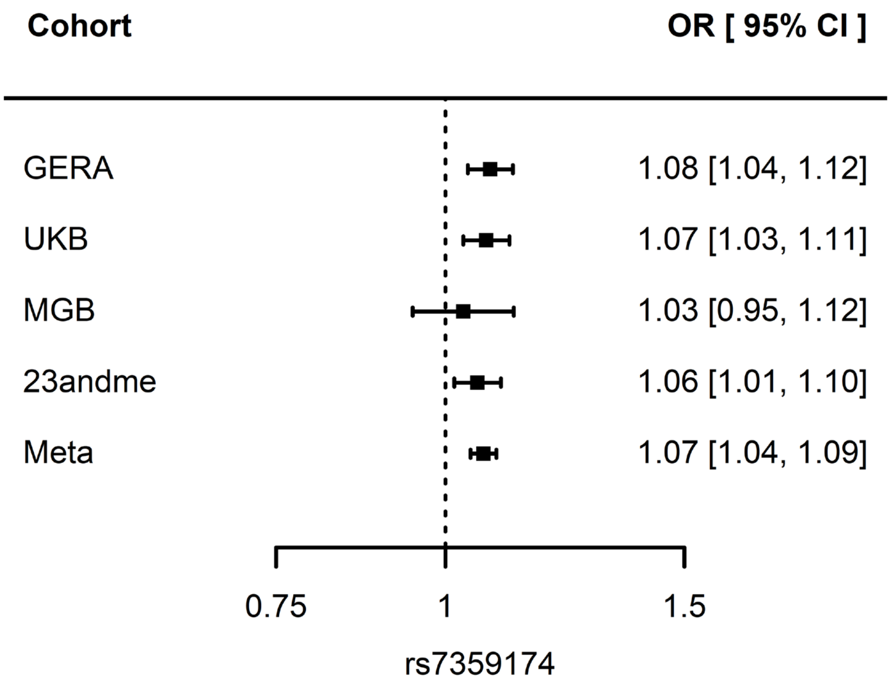
Cohort
OR [95% CI]
GERA
1.08 [1.04, 1.12]
UKB
1.07 [1.03, 1.11]
MGB
1.03 [0.95, 1.12]
23andme
1.06 [1.01, 1.10]
Meta
1.07 [1.04, 1.09]
0.75
1
1.5
rs7359174

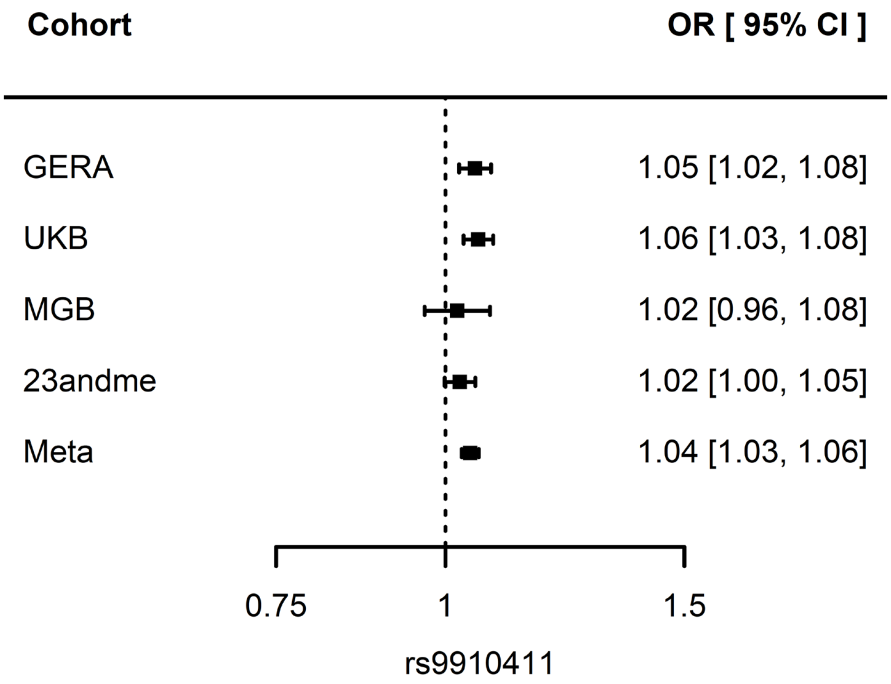
Cohort
OR [95% CI]
GERA
1.05 [1.02, 1.08]
UKB
1.06 [1.03, 1.08]
MGB
1.02 [0.96, 1.08]
23andme
1.02 [1.00, 1.05]
Meta
1.04 [1.03, 1.06]
0.75
1
1.5
rs9910411

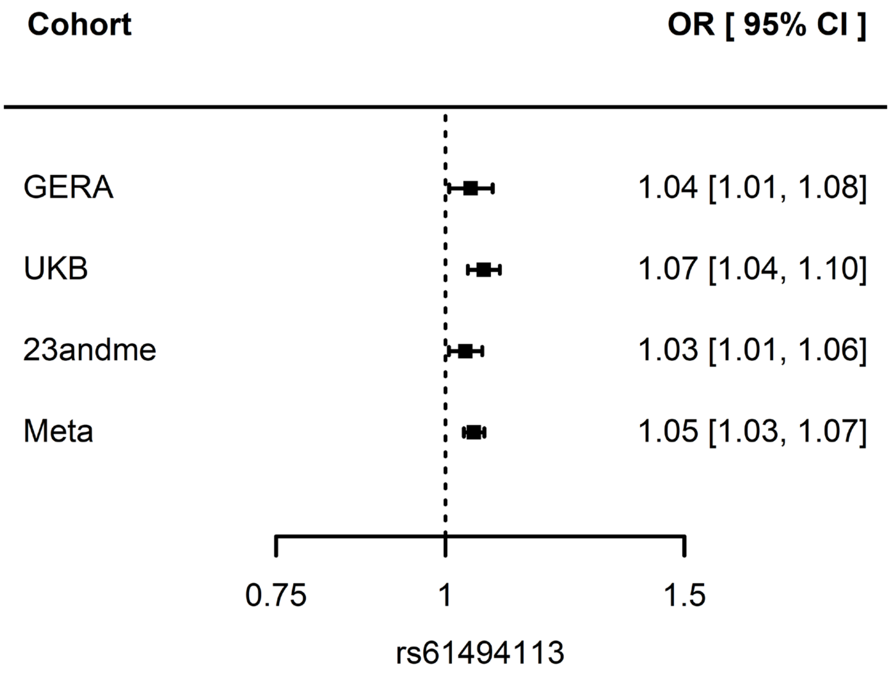
Cohort
OR [95% CI]
GERA
1.04 [1.01, 1.08]
UKB
1.07 [1.04, 1.10]
23andme
1.03 [1.01, 1.06]
Meta
1.05 [1.03, 1.07]
0.75
1
1.5
rs61494113

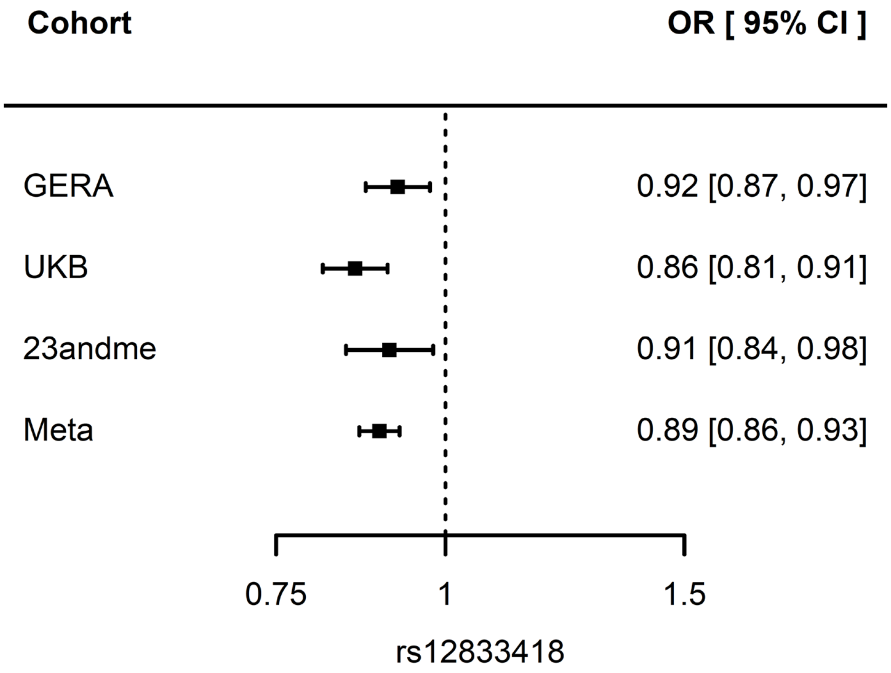
Cohort
OR [95% CI]
GERA
0.92 [0.87, 0.97]
UKB
0.86 [0.81, 0.91]
23andme
0.91 [0.84, 0.98]
Meta
0.89 [0.86, 0.93]
0.75
1
1.5
rs12833418

Supplementary Figure 5. QQ plot and genomic inflation factor (λ) observed for the Hispanic/Latino ancestry GWA meta-analysis of BCC

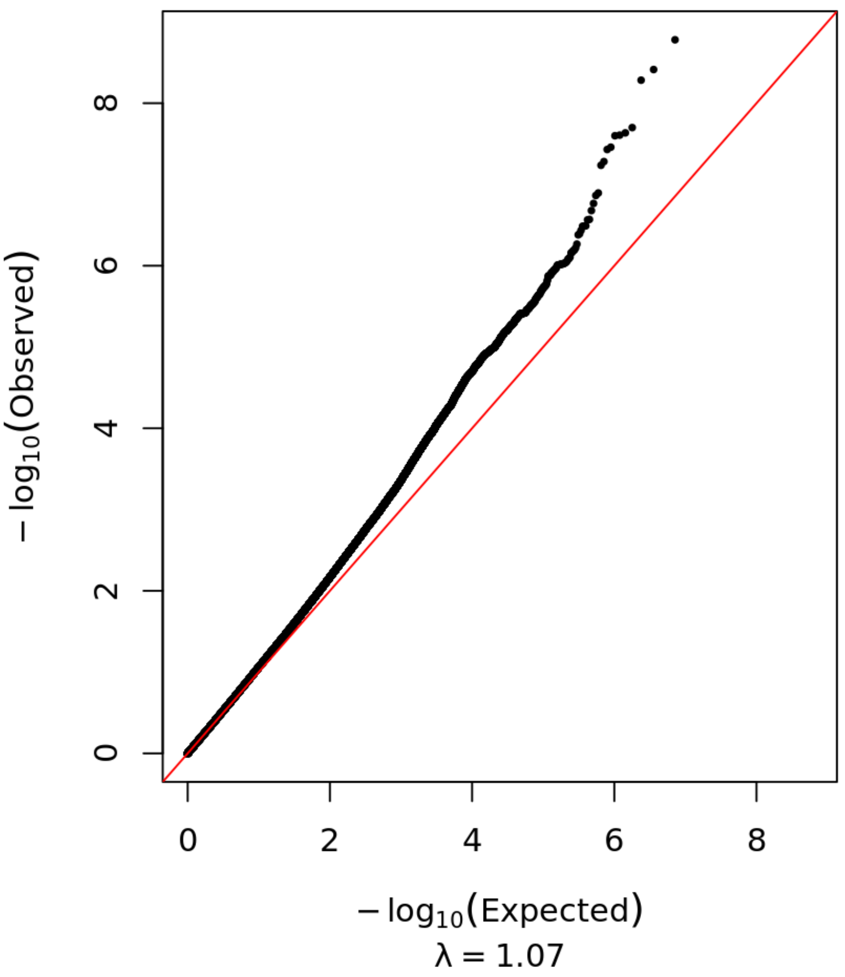

Supplementary Figure 6. Manhattan plot of the Hispanic/Latino ancestry GWA meta-analysis of BCC. The y-axis represents the -$\log_{10}$(*P*-value); all *P*-values derived from logistic regression model are two-sided. The red dotted line represents the threshold of *P*=5x10^{-8} which is the commonly accepted threshold of adjustments for multiple comparisons in GWAS.

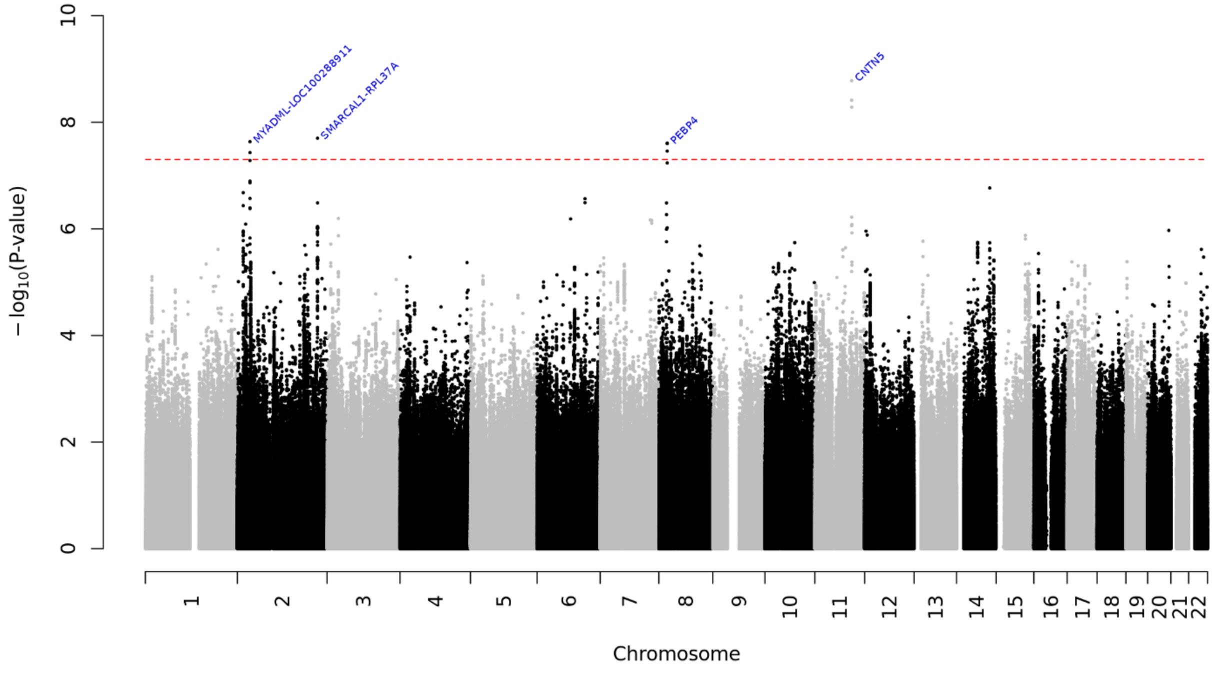

Supplementary Figure 7. Locus Zoom plots of regions identified in the Hispanic/Latino GWA meta-analysis of BCC or showing association with BCC at Bonferroni level of significance. Results are from the Hispanic/Latino ancestry GWA meta-analysis combining- data from the GERA and MGB cohorts.

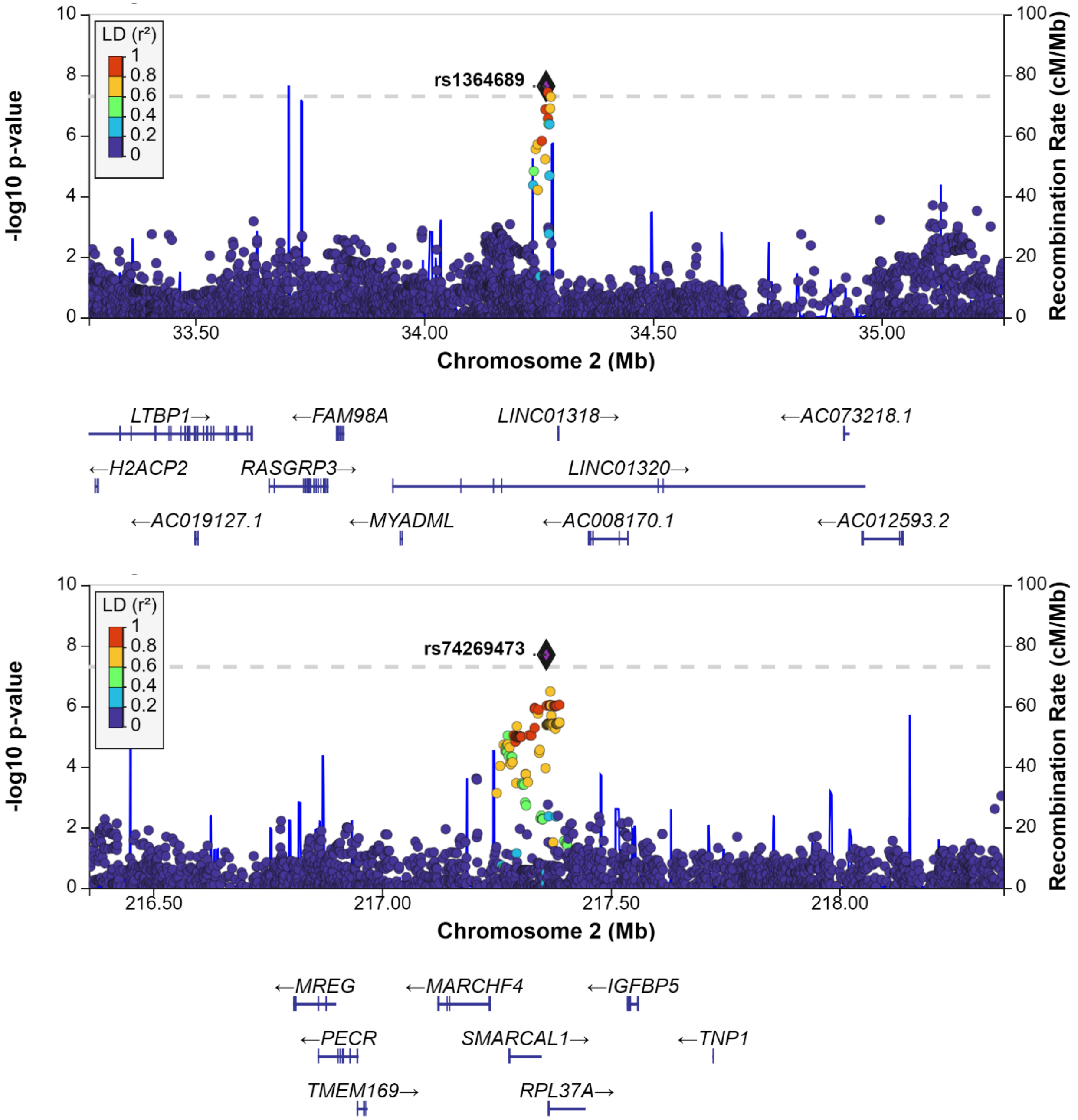

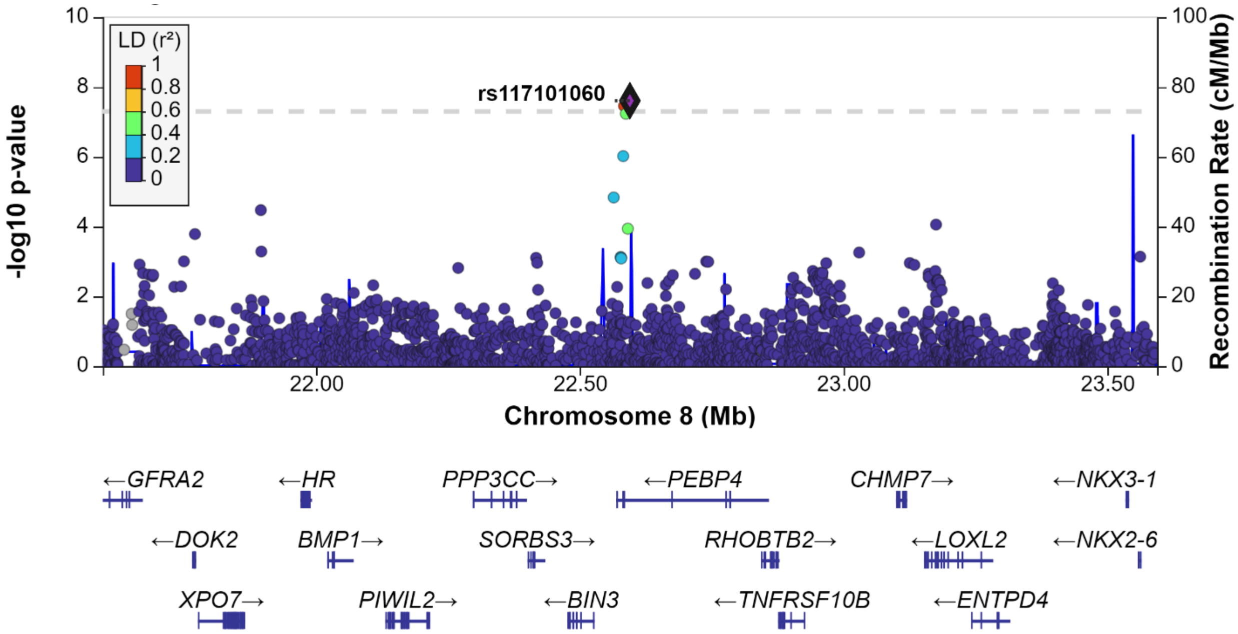

LD (r²)
1
0.8
0.6
0.4
0.2
0
rs117101060
-log10 p-value
Recombination Rate (cM/Mb)
22.00
22.50
23.00
23.50
Chromosome 8 (Mb)
←GFRA2
←HR
PPP3CC→
←PEBP4
CHMP7→
←NKX3-1
←DOK2
BMP1→
SORBS3→
RHOBTB2→
←LOXL2
←NKX2-6
XPO7→
PIWIL2→
←BIN3
←TNFRSF10B
←ENTPD4

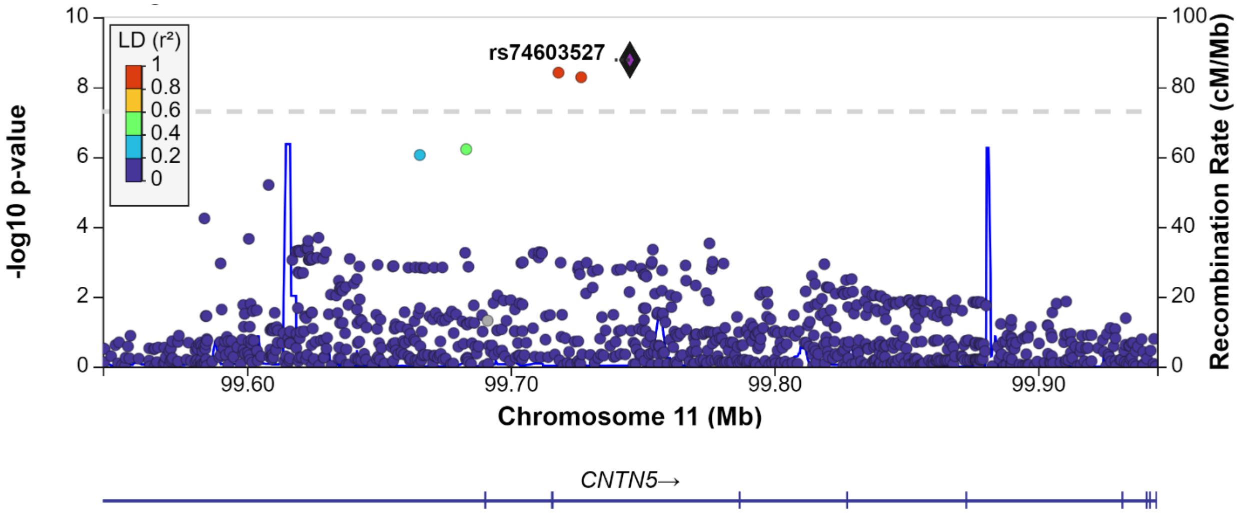

LD (r²)
1
0.8
0.6
0.4
0.2
0
rs74603527
-log10 p-value
Recombination Rate (cM/Mb)
99.60
99.70
99.80
99.90
Chromosome 11 (Mb)
CNTN5→

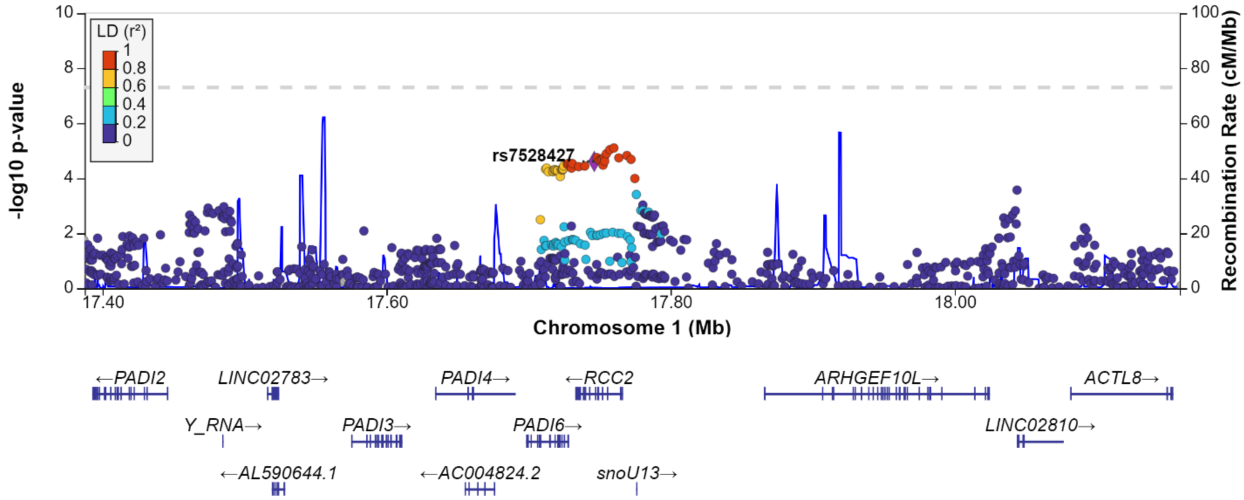
LD (r²)
1
0.8
0.6
0.4
0.2
0
rs7528427
-log10 p-value
Recombination Rate (cM/Mb)
Chromosome 1 (Mb)
17.40
17.60
17.80
18.00
←PADI2
LINC02783→
PADI4→
←RCC2
ARHGEF10L→
ACTL8→
Y_RNA→
PADI3→
PADI6→
LINC02810→
←AL590644.1
←AC004824.2
snoU13→

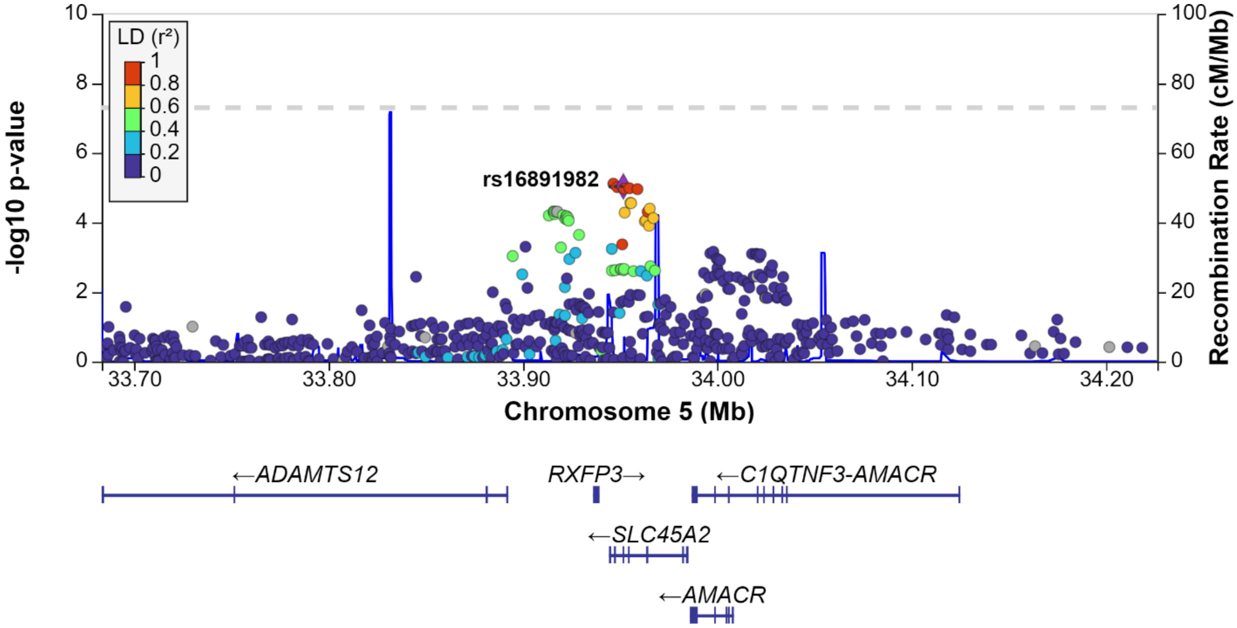
LD (r²)
1
0.8
0.6
0.4
0.2
0
rs16891982
-log10 p-value
Recombination Rate (cM/Mb)
Chromosome 5 (Mb)
33.70
33.80
33.90
34.00
34.10
34.20
←ADAMTS12
RXFP3→
←C1QTNF3-AMACR
←SLC45A2
←AMACR

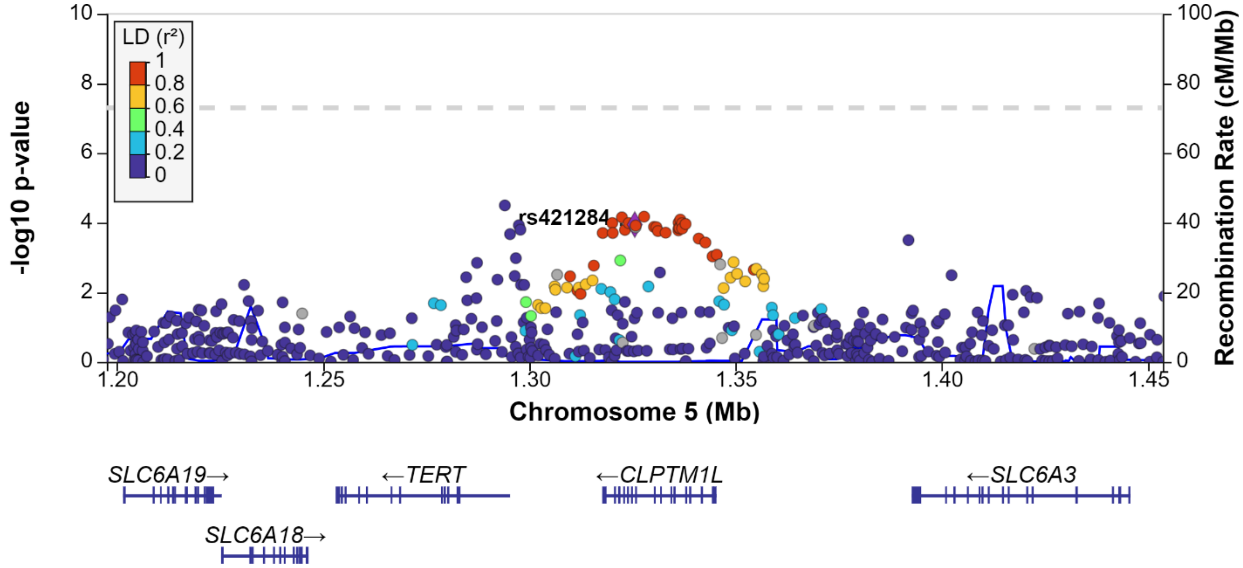

LD (r²)
1
0.8
0.6
0.4
0.2
0
rs421284
-log10 p-value
Recombination Rate (cM/Mb)
Chromosome 5 (Mb)
1.20
1.25
1.30
1.35
1.40
1.45
SLC6A19→
←TERT
←CLPTM1L
←SLC6A3
SLC6A18→

Supplementary Figure 8. Receiver operating characteristic curves for BCC predictions in the GERA Hispanic/Latino sample, using polygenic risk scores derived from the European ancestry GWA meta-analysis of BCC. The two different colors represent two different curves for each of the different EUR-derived PRSs of BCC: blue, PRS included clumped independent SNPs at *P*-value threshold < 5.0×10^{-8}; and red, PRS included clumped independent SNPs at *P*-value threshold < 1.0×10^{-6}. AUC values and standard deviations are reported to the legend. Abbreviation: AUC, area under the curve.

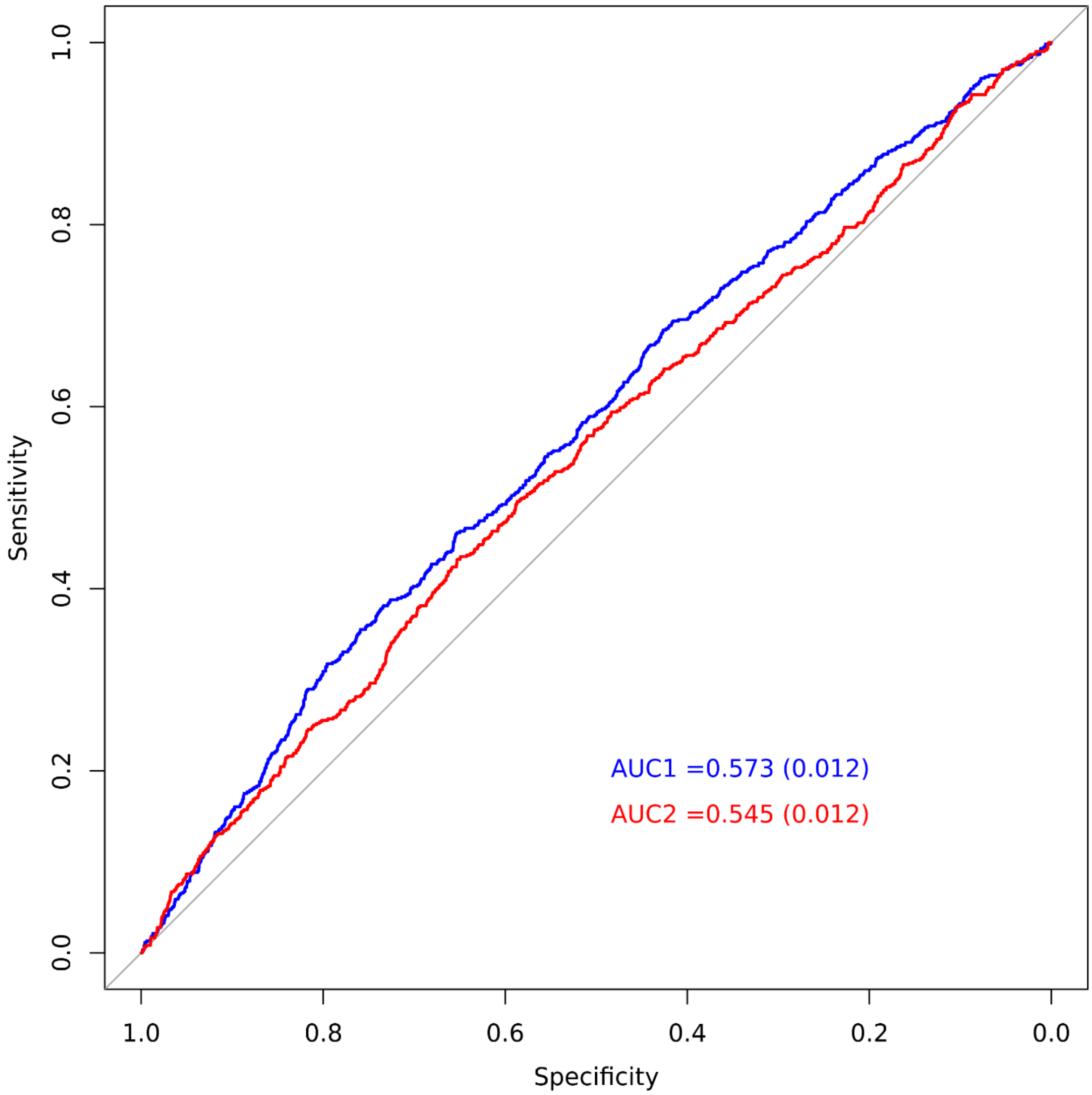

Supplementary Figure 9. QQ plot and genomic inflation factor (λ) observed for the multi-ancestry GWA meta-analysis of BCC

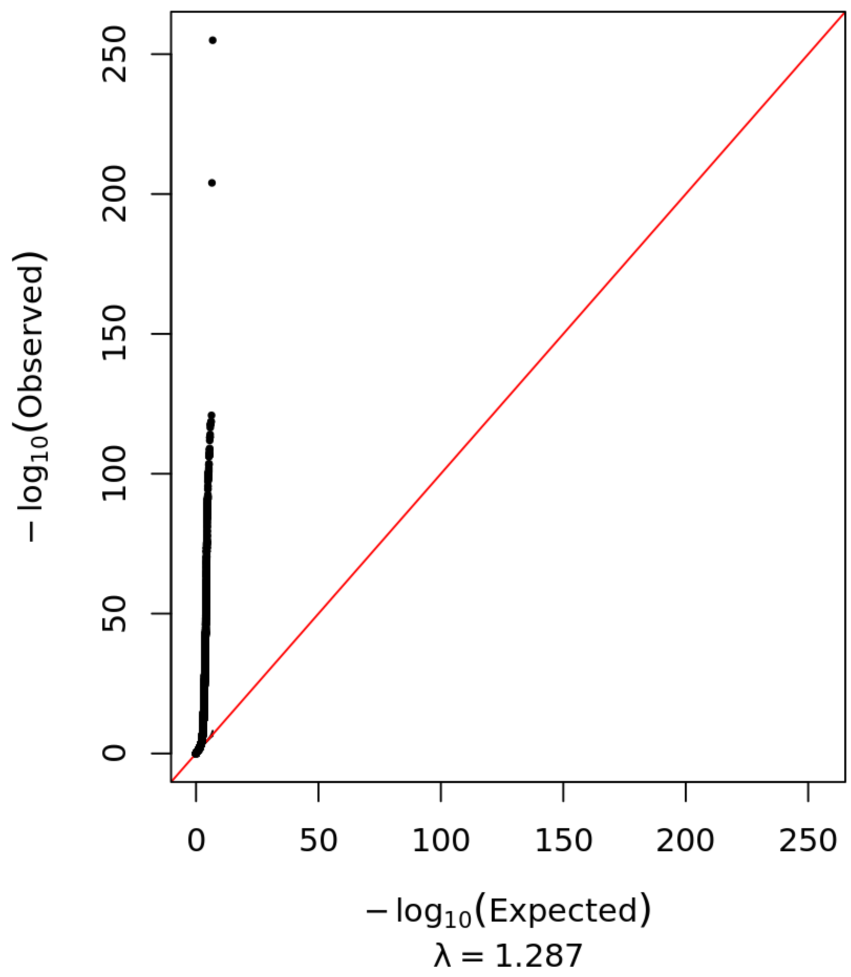

Supplementary Figure 10. Locus Zoom plots of additional novel regions identified in the multi-ancestry GWA meta-analysis of BCC.

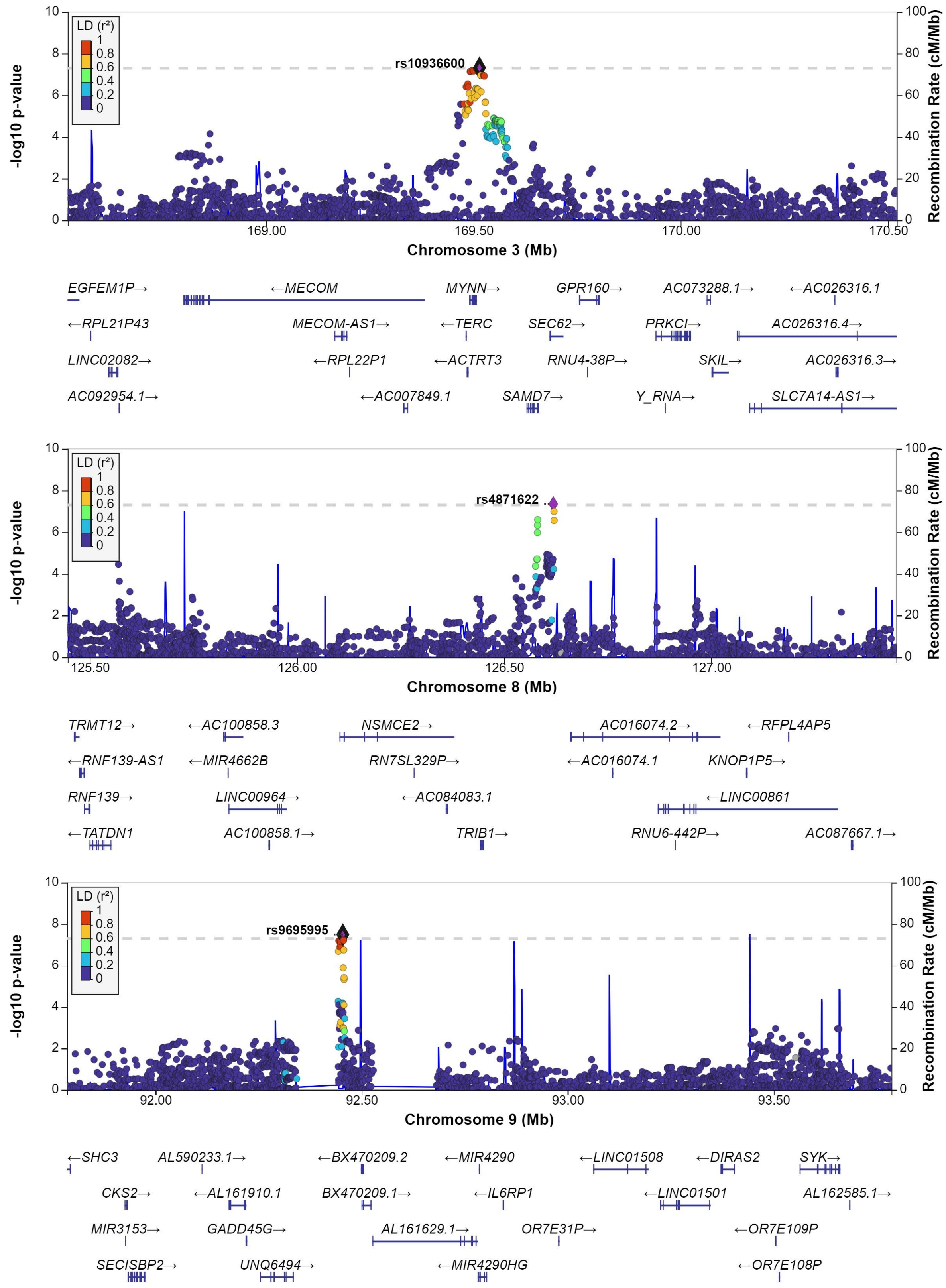

Supplementary Figure 11. Correlation of effect sizes across populations (European ancestry vs. Hispanic/Latino) for the lead 116 BCC-associated lead SNPs identified in the European ancestry GWA meta-analysis of BCC. The error bars correspond to the 95% CIs.

a. EUR (GERA+MGB+UKB+23andMe) vs. LAT (GERA+MGB)

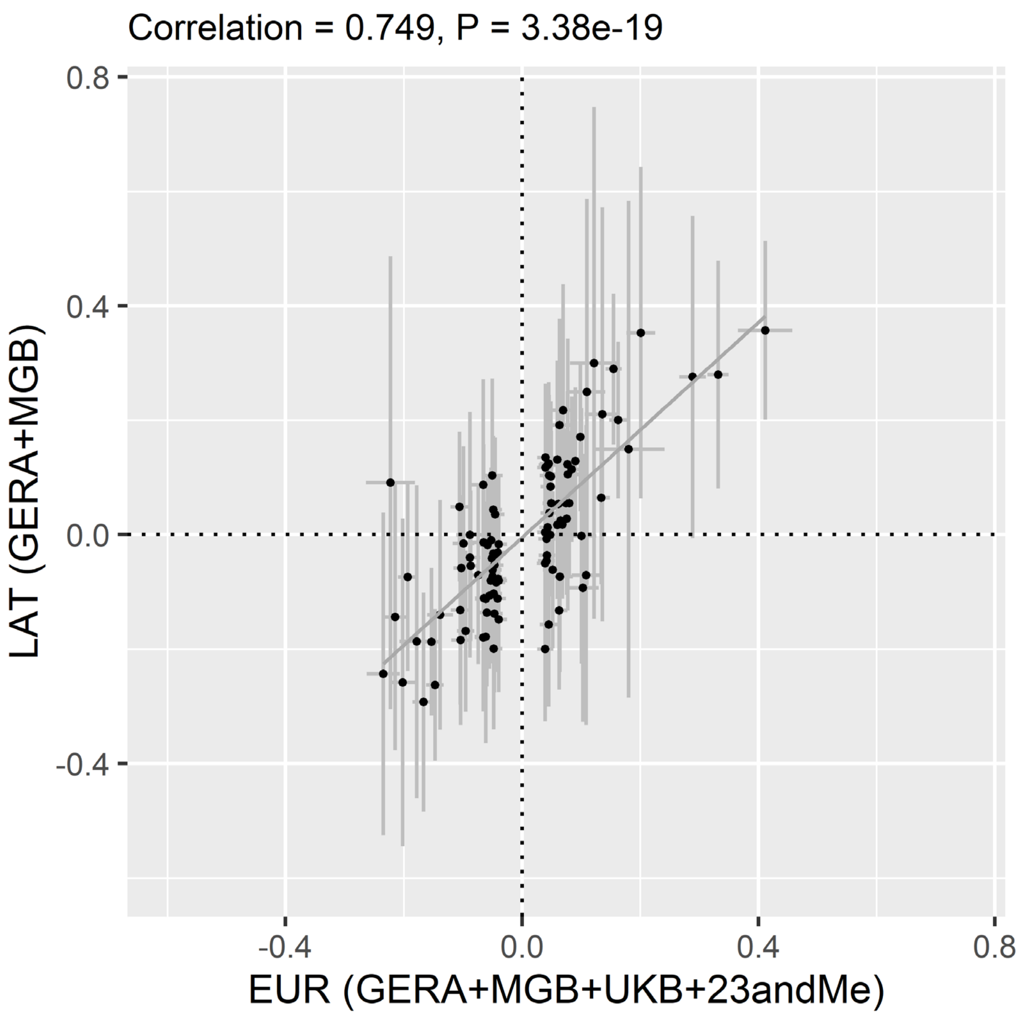

b. EUR (GERA) vs. LAT (GERA)

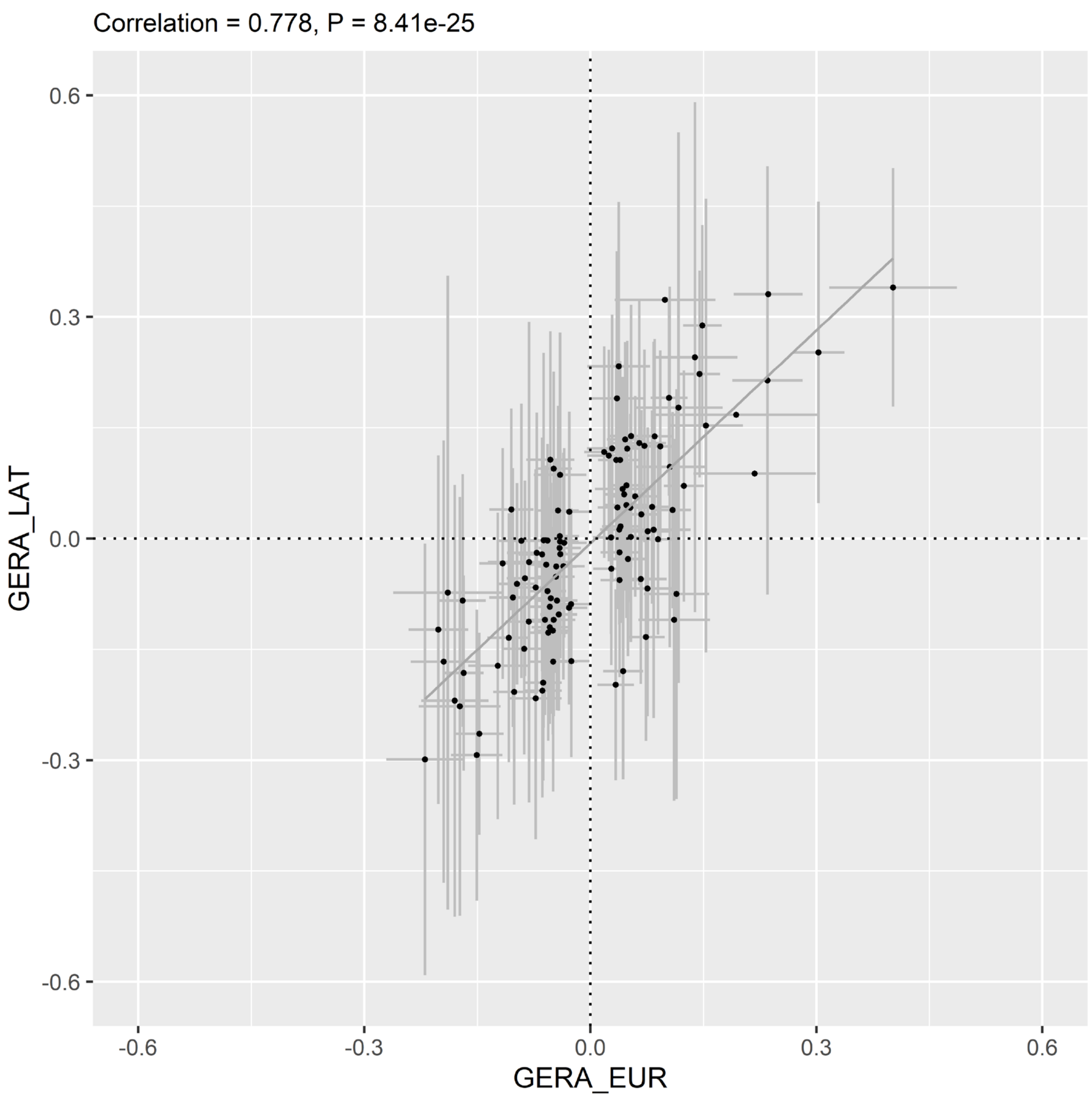

Supplementary Figure 12. QQ plot and genomic inflation factor (λ) observed for the European ancestry GWA meta-analysis of SCC

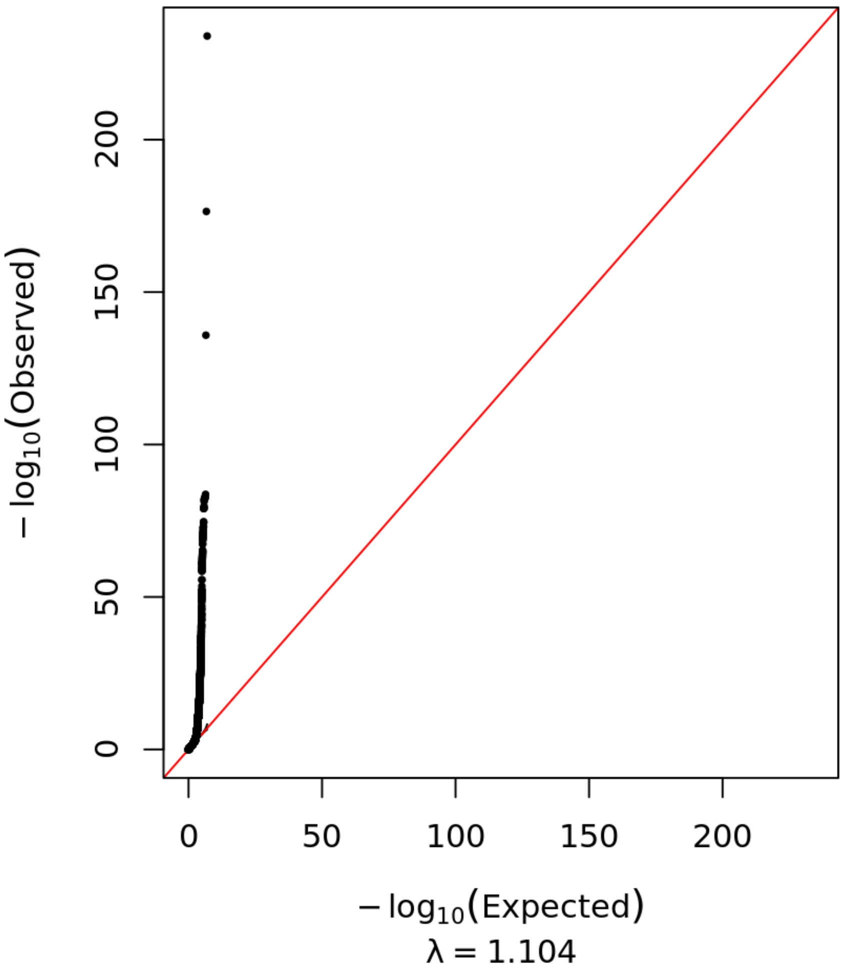

Supplementary Figure 13. Manhattan plot of the European ancestry GWA meta-analysis of SCC. The y-axis represents the -$\log_{10}$(*P*-value); all *P*-values derived from logistic regression model are two-sided. The red dotted line represents the threshold of P=5x10^{-8} which is the commonly accepted threshold of adjustments for multiple comparisons in GWAS. All loci have been previously reported.

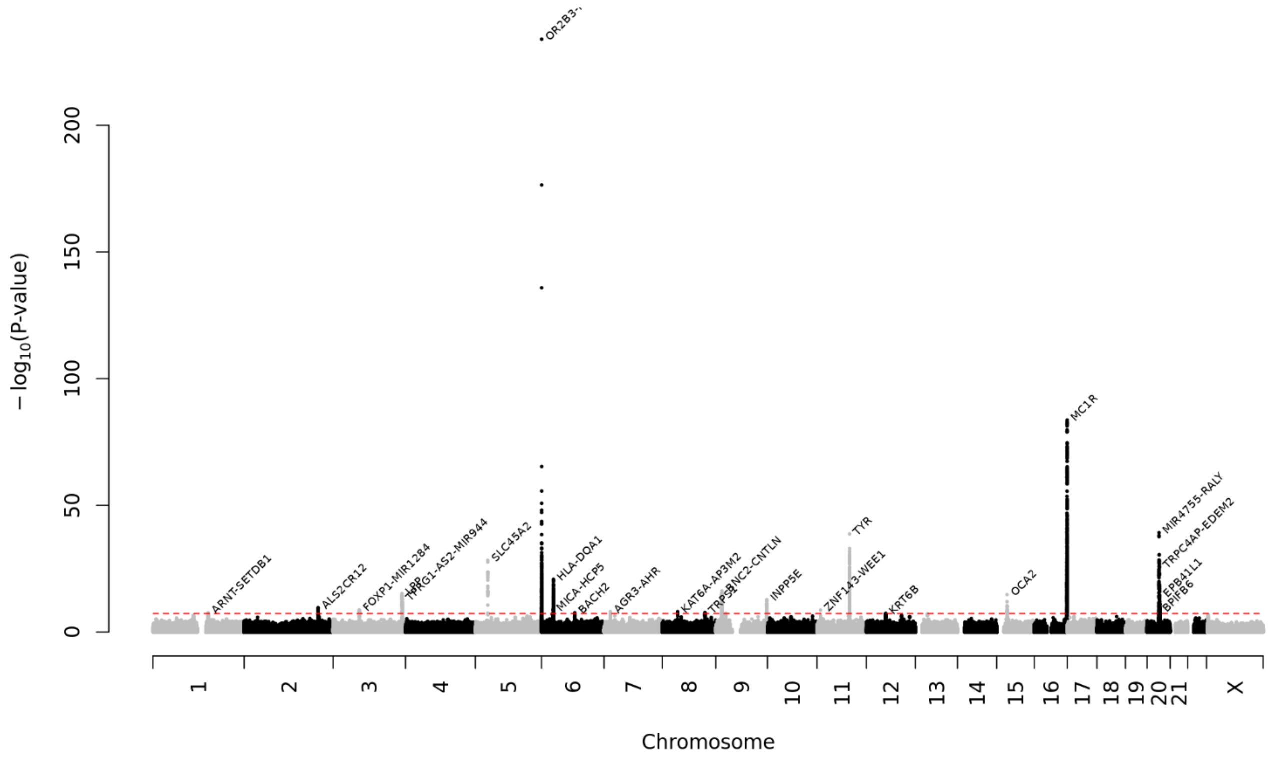

Supplementary Figure 14. Genome-wide genetic correlations between BCC and SCC across cohorts.

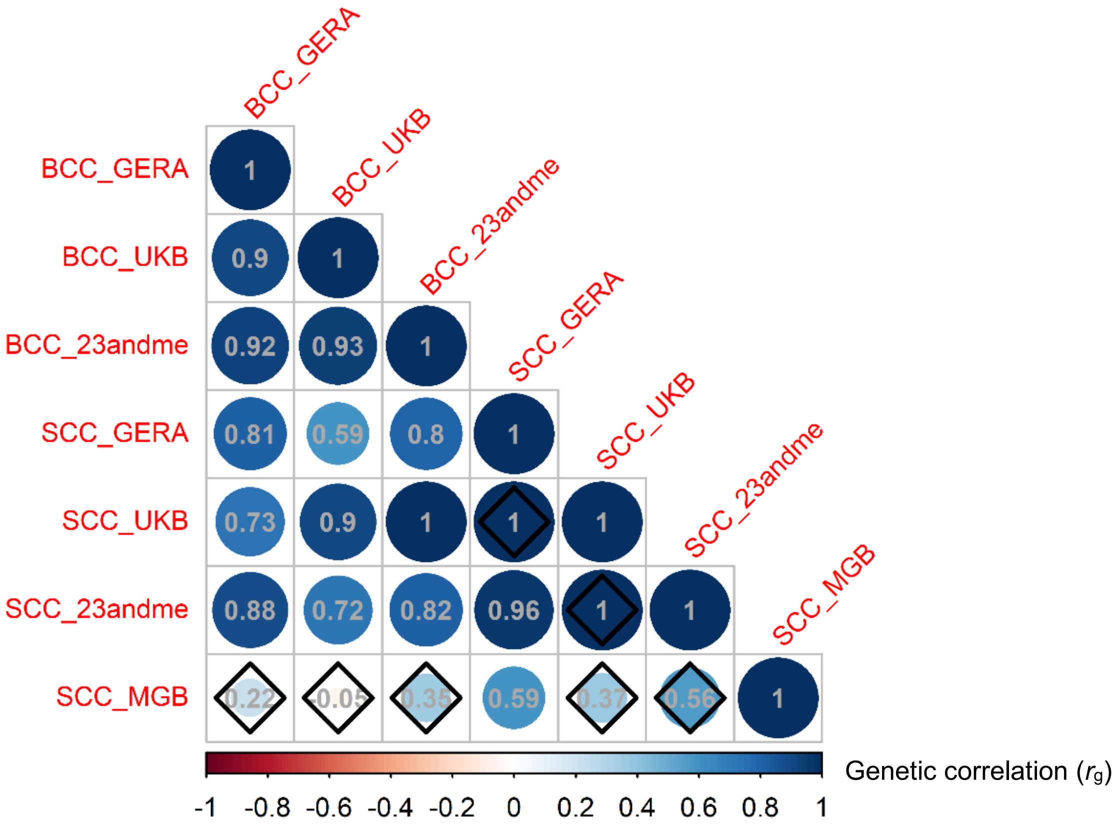

Note: summary of genetic correlation results for BCC_MGB are missing, as the input result for the concerned sample has very low heritability (h^2), suggesting not enough polygenic signals.

Supplementary Notes

Supplementary Note 1: Details for UKB on phenotyping

In **UKB**, BCC or SCC cases were defined as participants with an ICD-9 or ICD-10 diagnosis code for BCC or SCC and based on histology data (e.g. field ID: 40011). Especially, BCC/SCC cases were defined using the following codes:

BCC case definition: have at least one ICD10 or ICD9 diagnosis

#ICD10 invasive

anything starting C44 (e.g. C447, C442 etc) in f.40006.0.0 PLUS 8090-8094 or 8097-98 in f.40011.0.0 or
anything starting C44 (e.g. C447, C442 etc) in f.40006.1.0 PLUS 8090-8094 or 8097-98 in f.40011.1.0 or
anything starting C44 (e.g. C447, C442 etc) in f.40006.2.0 PLUS 8090-8094 or 8097-98 in f.40011.2.0 or
...etc down to x=9 in f.40006.x.0 and f.40011.x.0

#ICD9 invasive

anything starting 173 (e.g. 1730, 1731 etc) in f.40013.0.0 PLUS 8090-8094 or 8097-98 in f.40011.0.0 or
anything starting 173 (e.g. 1730, 1731 etc) in f.40013.1.0 PLUS 8090-8094 or 8097-98 in f.40011.1.0 or
anything starting 173 (e.g. 1730, 1731 etc) in f.40013.2.0 PLUS 8090-8094 or 8097-98 in f.40011.2.0 or
...etc down to x=10 in f.40013.x.0 and f.40011.x.0

SCC case definition: have at least one ICD10 or ICD9 diagnosis

#ICD10 invasive

anything starting C44 (e.g. C447, C442 etc) in f.40006.0.0 PLUS 8070-8076 or 8078 in f.40011.0.0 or
anything starting C44 (e.g. C447, C442 etc) in f.40006.1.0 PLUS 8070-8076 or 8078 in f.40011.1.0 or
anything starting C44 (e.g. C447, C442 etc) in f.40006.2.0 PLUS 8070-8076 or 8078 in f.40011.2.0 or
...etc down to x=9 in f.40006.x.0 and f.40011.x.0

#ICD9 invasive

anything starting 173 (e.g. 1730, 1731 etc) in f.40013.0.0 PLUS 8070-8076 or 8078 in f.40011.0.0 or
anything starting 173 (e.g. 1730, 1731 etc) in f.40013.1.0 PLUS 8070-8076 or 8078 in f.40011.1.0 or
anything starting 173 (e.g. 1730, 1731 etc) in f.40013.2.0 PLUS 8070-8076 or 8078 in f.40011.2.0 or
...etc down to x=10 in f.40013.x.0 and f.40011.x.0

Supplementary Note 2: Details for each cohort on genotyping, imputation, and quality control

Samples for each cohort (GERA, MGB, UK Biobank, and 23andMe Research cohort) were collected with informed consent and were genotyped on a variety of commercial arrays, as detailed before[1-7].

GERA

The GERA cohort consists of 110,266 adults who consented to the Research Program on Genes, Environment, and Health at Kaiser Permanente Northern California (KPNC). GERA participants' DNA samples were extracted from Oragene kits (DNA Genotek Inc., Ottawa, ON, Canada) at KPNC and genotyped at the Genomics Core Facility of the University of California, San Francisco (UCSF). DNA samples were genotyped at over 665,000 genetic markers on four ethnic-specific Affymetrix Axiom arrays (Affymetrix, Santa Clara, CA, USA) optimized for European, Latino, East Asian, and African American individuals[1,2]. Genotype quality control (QC) procedures and imputation were conducted on an array-wise basis[8], after an updated genotyping algorithm with an advanced normalization step specifically for SNPs in batches not recommended or flagged by the outlier plate detector than has previously been done. Subsequently, variants were excluded if: >3 clusters were identified; the number of batches was <38/42 (EUR array), <3/5 (AFR), <3/6 (EAS), or <7/9 (LAT); and the ratio of expected allele frequency variance across packages was <100 (EUR), <50 (AFR), <100 (EAS), <200 (LAT). On the EUR array, variants were additionally excluded if heterozygosity >.52 or <.02, and if an association test between Reagent kit v1.0 and v2.0 had $P<10^{-4}$. Imputation was done by array, and we additionally removed variants with call rates <90%. Genotypes were then pre-phased with Eagle[9] v2.3.2, and then imputed with Minimac3[10] v2.1.1, twice, using two reference panels, in a manner almost identical to that done in the UKB[6], and described previously[11]. In detail, the two reference panels included (1) the EGA release of the Haplotype Reference Consortium (n=27,165; no indels)[12] and (2) the 1000 Genomes Project Phase 3 (N=2,404; e.g., indels)[13]. The first reference panel (1) uses a larger sample size (including all the individuals in (2)) and should theoretically impute better, but it is only available at non-indels due to harmonization issues; the second reference panel (2) includes, e.g., indels, which are not present in the first panel, but at a smaller sample size. Thus, we imputed using both reference panels, but if a variant was in both (1) and (2), we used that which should have imputed better, ie., (1). As above-mentioned imputation in GERA was almost identical to that of UKB as has been described[6].

MGB

The MGB Biobank is an extensive integrated database containing clinical data from MGB HealthCare for ~100,000 consented patients and genomic data for over 35,000 participants[5]. MGB samples were genotyped using three versions of SNP array offered by Illumina (Illumina, Inc., San Diego, CA), including 1) Multi-Ethnic Genotyping Array (MEGA) array including 1,416,020 SNPs, 2) Expanded Multi-Ethnic Genotyping Array (MEGA Ex) array including 1,741,376 SNPs, 3) Multi-Ethnic Global (MEG) array including 1,778,953 SNPs, and 4) Global Screening Array (GSA). The Genome Reference Consortium Human genome build 37 (GRCh37) was used in annotating variants. Imputation was performed using the Michigan Imputation Server that uses Minimac3[10]. MGB Biobank uses the HRC reference panel (Version r1.1 2016) for imputation. This HRC panel consists of 64,940 haplotypes of predominantly European ancestry. Haplotype phasing was performed using SHAPE-IT[14]. A principal

component analysis (PCA) was applied to characterize population structure and exclude racial outliers. QC steps of genotyped data were conducted for the PCA. Briefly, any variants with a SNP call rate <98% and MAF < 0.01, as well as any subjects with sample call rate < 98%, a discrepancy between the reported and predicted sex, evidence of an excess of homozygosity, or related or duplicated subjects (identity-by-descent [IBD] > 0.2) were excluded from the PCA. For the genome-wide association analyses, imputed SNPs were used. Only common variants of four arrays (MEG, MEGA, MEGA EX, GSA-A) were included in further analyses after conducting QC for each array. Autosomal biallelic SNPs with an info score > 0.8 (high-quality imputed SNPs), call rates > 95%, and MAF > 1% were retained for the association analyses.

UK Biobank

The UK Biobank is a large prospective study following the health of approximately 500,000 participants resident in the UK aged between 40 and 69 years-old at the baseline recruitment visit[7]. Genotyping and imputation of UK Biobank samples were carried out by members of the UK Biobank team, and more information on participant recruitment, genotyping and imputation have been published elsewhere[6,7]. Briefly, individuals were genotyped using the UK BiLEVE Axiom and UKB Axiom Arrays[6]. After genetic quality control procedures, imputation to the two reference panels (1) the full HRC (n=32,488; no indels) and (2) a merger of 1000 Genomes Phase 3 and the UK10K (n=6,285; indels) was conducted as previously described[6].

23andMe Research Cohort

Genotyping of the 23andMe samples was conducted using four different arrays (V1, V2, V3, V4), that included custom variants and content from the Illumina HumanHap550+ BeadChip, and Illumina OmniExpress + BeadChip arrays. Imputation to version 3 release of the 1000 Genomes reference haplotypes[15] was conducted using Minimac2[4,16].The full GWAS descriptions, including details on data collection, genotyping, imputation and quality control) of 23andMe research cohort as a discovery GWAS (for both BCC and SCC) have been previously reported[3,4].

Supplementary References

1 Hoffmann, T. J. *et al.* Next generation genome-wide association tool: design and coverage of a high-throughput European-optimized SNP array. *Genomics* **98**, 79-89 (2011). https://doi.org/10.1016/j.ygeno.2011.04.005

2 Hoffmann, T. J. *et al.* Design and coverage of high throughput genotyping arrays optimized for individuals of East Asian, African American, and Latino race/ethnicity using imputation and a novel hybrid SNP selection algorithm. *Genomics* **98**, 422-430 (2011). https://doi.org/10.1016/j.ygeno.2011.08.007

3 Chahal, H. S. *et al.* Genome-wide association study identifies novel susceptibility loci for cutaneous squamous cell carcinoma. *Nat Commun* **7**, 12048 (2016). https://doi.org/10.1038/ncomms12048

4 Chahal, H. S. *et al.* Genome-wide association study identifies 14 novel risk alleles associated with basal cell carcinoma. *Nat Commun* **7**, 12510 (2016). https://doi.org/10.1038/ncomms12510

5 Weiss, S. T. & Shin, M. S. Infrastructure for Personalized Medicine at Partners HealthCare. *J Pers Med* **6** (2016). https://doi.org/10.3390/jpm6010013

6 Bycroft, C. *et al.* The UK Biobank resource with deep phenotyping and genomic data. *Nature* **562**, 203-209 (2018). https://doi.org/10.1038/s41586-018-0579-z

7 Sudlow, C. *et al.* UK biobank: an open access resource for identifying the causes of a wide range of complex diseases of middle and old age. *PLoS Med* **12**, e1001779 (2015). https://doi.org/10.1371/journal.pmed.1001779

8 Kvale, M. N. *et al.* Genotyping Informatics and Quality Control for 100,000 Subjects in the Genetic Epidemiology Research on Adult Health and Aging (GERA) Cohort. *Genetics* **200**, 1051-1060 (2015). https://doi.org/10.1534/genetics.115.178905

9 Loh, P. R. *et al.* Reference-based phasing using the Haplotype Reference Consortium panel. *Nat Genet* **48**, 1443-1448 (2016). https://doi.org/10.1038/ng.3679

10 Das, S. *et al.* Next-generation genotype imputation service and methods. *Nat Genet* **48**, 1284-1287 (2016). https://doi.org/10.1038/ng.3656

11 Hoffmann, T. J. *et al.* A large genome-wide association study of QT interval length utilizing electronic health records. *Genetics* **222** (2022). https://doi.org/10.1093/genetics/iyac157

12 McCarthy, S. *et al.* A reference panel of 64,976 haplotypes for genotype imputation. *Nat Genet* **48**, 1279-1283 (2016). https://doi.org/10.1038/ng.3643

13 Birney, E. & Soranzo, N. Human genomics: The end of the start for population sequencing. *Nature* **526**, 52-53 (2015). https://doi.org/10.1038/526052a

14 Delaneau, O., Marchini, J. & Zagury, J. F. A linear complexity phasing method for thousands of genomes. *Nat Methods* **9**, 179-181 (2011). https://doi.org/10.1038/nmeth.1785

15 Genomes Project, C. *et al.* A map of human genome variation from population-scale sequencing. *Nature* **467**, 1061-1073 (2010). https://doi.org/10.1038/nature09534

16 Fuchsberger, C., Abecasis, G. R. & Hinds, D. A. minimac2: faster genotype imputation. *Bioinformatics* **31**, 782-784 (2015). https://doi.org/10.1093/bioinformatics/btu704
