# Supplementary material for: Multi-ancestry genome-wide meta-analysis identifies novel basal cell carcinoma loci and shared genetic effects with squamous cell carcinoma
Source: Commun Biol. 2024 Jan 5;7:33. doi: 10.1038/s42003-023-05753-7 (PMC10770328; doi:10.1038/s42003-023-05753-7)
Supplement: Supplementary file 3 — Description of Additional Supplementary Files [file 42003_2023_5753_MOESM3_ESM.pdf]

Description of Additional Supplementary Files

File name: Supplementary Data 1
Description: BCC and SCC study cohorts included in the GWA analyses.

File name: Supplementary Data 2
Description: BCC-associated loci identified in the European ancestry GWA meta-analysis and results in each cohort.

File name: Supplementary Data 3
Description: BCC-associated variants identified in the conditional and joint multiple-SNP (COJO) analysis conducted on the European ancestry meta-analysis results.

File name: Supplementary Data 4
Description: Look-up of the 116 BCC associated loci identified in the European ancestry meta-analysis in the Hispanic/Latino GWA meta-analysis of BCC.

File name: Supplementary Data 5
Description: BCC-associated loci identified in the Hispanic/Latino GWA meta-analysis.

File name: Supplementary Data 6
Description: BCC-associated loci identified in the multi-ancestry GWA meta-analysis.

File name: Supplementary Data 7
Description: SCC-associated loci identified in the European ancestry GWA meta-analysis.

File name: Supplementary Data 8
Description: Association of the 116 BCC associated loci identified in the European ancestry meta-analysis with SCC risk.

File name: Supplementary Data 9
Description: LD score regression analysis results of individual GWAS datasets.

File name: Supplementary Data 10
Description: List of the 95% credible set of variants in each of the 116 loci identified in the European ancestry GWA meta-analysis of BCC.

File name: Supplementary Data 11
Description: VEGAS2 Gene-based association analysis results for BCC.

File name: Supplementary Data 12
Description: VEGAS2-Pathways association analysis results for BCC.

File name: Supplementary Data 13
Description: Prioritized drug targets for BCC.
