# Supplementary material for: Multi-ancestry genome-wide meta-analysis identifies novel basal cell carcinoma loci and shared genetic effects with squamous cell carcinoma
Source: Commun Biol. 2024 Jan 5;7:33. doi: 10.1038/s42003-023-05753-7 (PMC10770328; doi:10.1038/s42003-023-05753-7)
Supplement: Supplementary file 5 — Reporting Summary [file 42003_2023_5753_MOESM5_ESM.pdf]

nature portfolio

Corresponding author(s): Hélène Choquet

Last updated by author(s): Dec 21, 2023

Reporting Summary

Nature Portfolio wishes to improve the reproducibility of the work that we publish. This form provides structure for consistency and transparency in reporting. For further information on Nature Portfolio policies, see our Editorial Policies and the Editorial Policy Checklist.

Statistics

For all statistical analyses, confirm that the following items are present in the figure legend, table legend, main text, or Methods section.

n/a	Confirmed	
☐	☒	The exact sample size (*n*) for each experimental group/condition, given as a discrete number and unit of measurement
☐	☒	A statement on whether measurements were taken from distinct samples or whether the same sample was measured repeatedly
☐	☒	The statistical test(s) used AND whether they are one- or two-sided<br>*Only common tests should be described solely by name; describe more complex techniques in the Methods section.*
☐	☒	A description of all covariates tested
☐	☒	A description of any assumptions or corrections, such as tests of normality and adjustment for multiple comparisons
☐	☒	A full description of the statistical parameters including central tendency (e.g. means) or other basic estimates (e.g. regression coefficient) AND variation (e.g. standard deviation) or associated estimates of uncertainty (e.g. confidence intervals)
☐	☒	For null hypothesis testing, the test statistic (e.g. *F*, *t*, *r*) with confidence intervals, effect sizes, degrees of freedom and *P* value noted<br>*Give P values as exact values whenever suitable.*
☒	☐	For Bayesian analysis, information on the choice of priors and Markov chain Monte Carlo settings
☐	☒	For hierarchical and complex designs, identification of the appropriate level for tests and full reporting of outcomes
☐	☒	Estimates of effect sizes (e.g. Cohen's *d*, Pearson's *r*), indicating how they were calculated

Our web collection on statistics for biologists contains articles on many of the points above.

Software and code

Policy information about availability of computer code

Data collection	Genotype analysis, quality control, phasing, and imputation analysis were performed on samples from the GERA cohort using the following softwares: Genotyping Console™ Software (Affymetrix) to perform genotype calling, quality control (QC) analysis, and sample or SNP filtering prior to downstream analysis. PLINK software v1.90 to perform additional QC analyses. Genotypes were then pre-phased with Eagle v2.3.2, and then imputed with Minimac3 v2.0.1. MGB samples were genotyped using three versions of SNP array offered by Illumina (Illumina, Inc., San Diego, CA), including 1) Multi-Ethnic Genotyping Array (MEGA) array including 1,416,020 SNPs, 2) Expanded Multi-Ethnic Genotyping Array (MEGA Ex) array including 1,741,376 SNPs, 3) Multi-Ethnic Global (MEG) array including 1,778,953 SNPs, and 4) Global Screening Array (GSA). Imputation was performed using the Michigan Imputation Server that uses Minimac3. All software programs employed are available for public use and no custom code was employed.
Data analysis	The GWAS analyses were performed using a recent approach accounting for relatedness that fits a whole-genome regression model, implemented in REGENIEv2.0.2. Genome-wide Complex Trait Analysis (GCTA) integrative tool was used to conduct a multi-SNP-based conditional & joint association analysis (COJO). A LD score regression was conducted, using the LDSC v1.0.1 command line tool (https://github.com/bulik/ldsc), to estimate the genome-wide genetic correlations between BCC and SCC across cohorts. The R programming language and software environment for statistical computing was used for calculating the two PRSs for BCC, as well as for conducting the logistic regression models ('glm') and for calculating AUC and generating the receiver operating characteristic curves ('ROCR'). CAVIARBF was used to prioritize genetic variants within the identified genomic regions. The Versatile Gene-based Association Study - 2 version 2 (VEGAS2v02) web platform was used to prioritize genes and biological pathways. We used Open Targets (http://genetics.opentargets.org), to search for drugs currently in use or in clinical trials for treating other skin cancer or systemic diseases that target the BCC risk genes identified in the current study.

For manuscripts utilizing custom algorithms or software that are central to the research but not yet described in published literature, software must be made available to editors and reviewers. We strongly encourage code deposition in a community repository (e.g. GitHub). See the Nature Portfolio guidelines for submitting code & software for further information.

Data

Policy information about availability of data

All manuscripts must include a data availability statement. This statement should provide the following information, where applicable:

- Accession codes, unique identifiers, or web links for publicly available datasets
- A description of any restrictions on data availability
- For clinical datasets or third party data, please ensure that the statement adheres to our policy

The GERA genotype data are available upon application to the KP Research Bank (https://researchbank.kaiserpermanente.org/). The MGB GWAS data are available by request with required approval from the Mass General Brigham Institutional Review board from https://www.massgeneralbrigham.org/en/research-and-innovation/for-researchers-and-collaborators. UK Biobank data, including BCC and SCC GWASs are available by request through the UK Biobank Access Management System https://www.ukbiobank.ac.uk/. The BCC and SCC GWAS results from 23andMe are available by request from https://www.23andme.com/. Restrictions apply to the availability of these data (please see https://research.23andme.com/dataset-access/), which were used under license for this study, and are not publicly available. Pathways or gene-sets were derived from the Biosystem's database which can be accessed through the following link (https://vegas2.qimrberghofer.edu.au/biosystems20160324.vegas2pathSYM).

Human research participants

Policy information about studies involving human research participants and Sex and Gender in Research.

Reporting on sex and gender	Sex was included as a covariate in all analyses.
Population characteristics	Age, sex, race/ethnicity, basal cell carcinoma diagnosis, cutaneous squamous cell carcinoma, history of cancer.<br>The Genetic Epidemiology Research in Adult Health and Aging (GERA) cohort consists of 110,266 adult men and women, 18 years and older, who are of non-Hispanic white, Hispanic/Latino, Asian or African American ethnicity.<br>The MGB Biobank is an extensive integrated database containing clinical data from MGB HealthCare for ~100,000 consented patients and genomic data for over 35,000 participants.<br>The UK Biobank (UKB) is a large prospective study following the health of approximately 500,000 participants resident in the UK aged between 40 and 69 years-old at the baseline recruitment visit.<br>23andMe Inc. (Mountain View, CA), a genetics company, provided free access to anonymized genetic and phenotypic information.
Recruitment	Participants from the GERA cohort are members of the Kaiser Permanente Northern California (KPNC) integrated health care delivery system, and provided self-reported information via the Research Program on Genes, Environment, and Health (RPGEH) survey.<br>Participants from MGB are members of the MGB HealthCare.<br>For UKB participants, demographic information and medical history were ascertained through touch-screen questionnaires. UKB participants also underwent a wide range of physical and cognitive assessments, including blood sampling.<br>23andMe gathers genetic information by genotyping sample material provided by its research participants; phenotypic information is collected via research participant responses to online surveys.
Ethics oversight	Samples for each cohort (GERA, MGB, UK Biobank, and 23andMe Research cohort) were collected with informed consent. For instance, for GERA, all study procedures were approved by the Institutional Review Board of the Kaiser Permanente Northern California Institutional Review Board. Written informed consent was obtained from all participants.<br>23andMe research participants provided informed consent to participate in research, in accord with 23andMe's human subjects protocol (reviewed and approved by Ethical and Independent Review Services, an AAHRPP accredited IRB).

Note that full information on the approval of the study protocol must also be provided in the manuscript.

Field-specific reporting

Please select the one below that is the best fit for your research. If you are not sure, read the appropriate sections before making your selection.

☒ Life sciences ☐ Behavioural & social sciences ☐ Ecological, evolutionary & environmental sciences

For a reference copy of the document with all sections, see nature.com/documents/nr-reporting-summary-flat.pdf

Life sciences study design

All studies must disclose on these points even when the disclosure is negative.

Sample size	In this study, we performed a European ancestry genome-wide association (GWA) meta-analysis and the first Hispanic/Latino ancestry GWA meta-analysis and meta-analyzed both in a multi-ancestry GWAS meta-analysis of BCC, totaling 50,531 BCC cases and 762,234 controls from four cohorts (GERA, Mass-General Brigham Biobank, UK Biobank, and 23andMe research cohort). While in the GERA cohort, BCC cases were identified from electronic pathology records using a validated SNOMED code-based algorithm, in the MGB and UK Biobank, BCC cases were

identified based on International Classification of Disease, Ninth (ICD-9) and/or Tenth (ICD-10) diagnosis codes, and in 23andMe research cohort, participants self-reported a history of BCC.

Data exclusions	In GERA, genotype quality control (QC) procedures and imputation were conducted on an array-wise basis, after an updated genotyping algorithm with an advanced normalization step specifically for SNPs in batches not recommended or flagged by the outlier plate detector than has previously been done. Subsequently, variants were excluded if: >3 clusters were identified; the number of batches was <38/42 (EUR array), <3/5 (AFR), <3/6 (EAS), or <7/9 (LAT); and the ratio of expected allele frequency variance across packages was <100 (EUR), <50 (AFR), <100 (EAS), <200 (LAT). On the EUR array, variants were additionally excluded if heterozygosity >.52 or <.02, and if an association test between Reagent kit v1.0 and v2.0 had P<10-4. Imputation was done by array, and we additionally removed variants with call rates <90%.<br>In MGB, any variants with a SNP call rate <98% and MAF < 0.01, as well as any subjects with sample call rate < 98%, a discrepancy between the reported and predicted sex, evidence of an excess of homozygosity, or related or duplicated subjects (identity-by-descent [IBD] > 0.2) were excluded.
Replication	In order to maximize power to detect novel BCC and SCC risk loci, we did not separate our sample into a separate discovery and replication set. Our results replicated most of previous known loci, especially from a recent large multi-trait genetic analysis that reported 78 risk loci for BCC. Our study also has important strengths, including the large sample size of up to 812,765 participants (50,531 BCC cases) from 4 different research studies (GERA, MGB, UKB, and 23andMe, Inc., research cohort)
Randomization	In the GERA cohort, BCC cases were identified from electronic pathology records. After excluding individuals with any evidence of metastatic BCCs (SNOMED codes M80906, M809061, M809063, M80909, M809092, M809093, M80946, M809492, M809493, M80960), our control group included all the non-cases after excluding individuals who had a current or prior cancer registry history of cancer, or benign or in-situ tumors, or had a self-reported cancer at the time of enrollment. Further, cSCC cases were defined as subjects whose pathology records were consistent with incident cSCC (invasive or in situ, excluding anogenital and mucosal SCCs); controls were subjects with no pathology records consistent with cSCC, and similar to our BCC control group, we excluded individuals who had a current or prior cancer registry history of cancer, or benign or in-situ tumors, or had a self-reported cancer at the time of enrollment. In MGB, BCC cases were defined as those with BCC diagnosis using ICD-10 (C44.01-C44.91) codes, while those without BCC diagnosis were considered controls. Further, cSCC cases were defined as those with cSCC diagnoses using International Classification of Disease (ICD), Ninth or Tenth Clinical Modification (CM) codes (ICD-9-CM: 173.0-173.9; ICD-10-CM: C44.0-C44.9) and were subsequently validated by electronic pathology reports review. Those without cSCC diagnosis were considered controls. In UKB, BCC or SCC cases were defined as participants with an ICD-9 or ICD-10 diagnosis code for BCC or SCC and based on histology data (e.g. field ID: 40011). As previously done, we excluded UKB participants who self-reported cancer at the time of enrollment and/or who had a current or prior cancer registry history of cancer, or benign or in-situ tumors, from the control groups. In the 23andMe research cohort, participants who self-reported a history of BCC or SCC cases were assigned to the BCC or SCC cases.
Blinding	Blinding was not relevant to our study. Participants were selected based on their BCC or SCC status.

Reporting for specific materials, systems and methods

We require information from authors about some types of materials, experimental systems and methods used in many studies. Here, indicate whether each material, system or method listed is relevant to your study. If you are not sure if a list item applies to your research, read the appropriate section before selecting a response.

Materials & experimental systems

n/a	Involved in the study	
☒	☐	Antibodies
☒	☐	Eukaryotic cell lines
☒	☐	Palaeontology and archaeology
☒	☐	Animals and other organisms
☒	☐	Clinical data
☒	☐	Dual use research of concern

Methods

n/a	Involved in the study	
☒	☐	ChIP-seq
☒	☐	Flow cytometry
☒	☐	MRI-based neuroimaging
